# Inherently chiral calix[4]arenes via oxazoline directed ortholithiation: synthesis and probe of chiral space

**DOI:** 10.3762/bjoc.10.291

**Published:** 2014-11-25

**Authors:** Simon A Herbert, Laura J van Laeren, Dominic C Castell, Gareth E Arnott

**Affiliations:** 1 Department of Chemistry and Polymer Science, Stellenbosch University, Private Bag X1, Matieland 7602, South Africa

**Keywords:** calix[4]arene, inherently chiral, ortholithiation, oxazoline, Tsuji–Trost

## Abstract

The diastereoselective oxazoline-directed lithiation of calix[4]arenes is reported with diastereoselective ratios of greater than 100:1 in some instances. Notably, it has been found that the opposite diastereomer can be accessed via this approach merely through the choice of an alkyllithium reagent. The inherently chiral oxazoline calix[4]arenes have also been preliminarily examined as ligands in the palladium-catalyzed Tsuji–Trost allylation reaction, returning results comparable to their planar chiral ferrocene counterparts pointing towards future application of these types of compounds.

## Introduction

Calix[4]arenes form an important class of supramolecules that have been widely studied since the seminal work of Gutsche in the 1970’s [1]. The reason for this interest stems from the calix[4]arene’s three dimensional bowl-shaped structure which imparts them with a number of interesting attributes and applications. These range from their use as molecular receptors [2–6], stationary phase modifiers in separation science [7–9], and as molecular storage vessels [10–13]. In the area of molecular recognition, the importance of chirality in the calixarene receptor has been shown to be significant [14–17]. With this in mind, chiral calixarenes have been synthesized, either by the incorporation of a chiral group into the calixarene scaffold, or by the introduction of asymmetry into the structure itself, creating chirality associated with form and termed inherent chirality [18]. Efforts to explore the properties of inherently chiral calixarenes have been hampered by the difficulty with which enantiomerically pure or enriched antipodes can be obtained. Until recently, no significantly asymmetric method for functionalizing calix[4]arenes had been developed, with all other efforts relying on various resolution techniques [16,19–26].

**Scheme 1:**
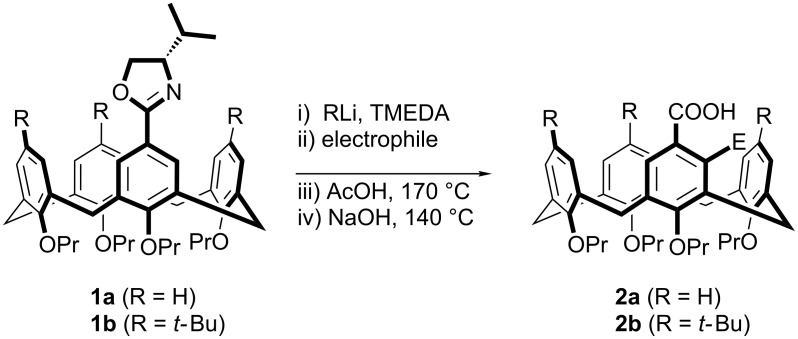
Synthesis of inherently chiral calix[4]arenes.

We, however, have reported that stereocontrolled *meta-*functionalization of a calix[4]arene is possible via chiral oxazoline-directed ortholithiation [27–28]. This has allowed for a range of functionalized inherently chiral calixarenes to be obtained, in good enantiomeric ratios (Scheme 1). We reasoned though that the diastereoselectivity of the reaction would be even better if a more bulky oxazoline was employed, i.e., using a *tert*-butyl group. Herein we report our results on the use of *tert-*butyloxazolines to synthesize inherently chiral calixarenes and the surprising reversal of diastereoselectivity obtained when a different alkyllithium was employed. We also wish to report a preliminary study on the application of inherently chiral calix[4]arenes in the classic asymmetric Tsuji–Trost allylation reaction.

## Results and Discussion

### Ortholithiation of *tert*-butyloxazoline calix[4]arenes

**Scheme 2:**
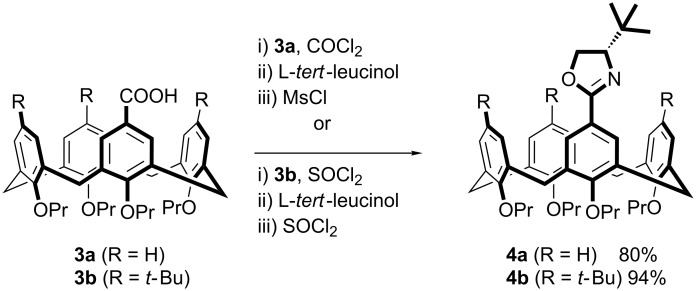
Synthesis of *tert*-butyloxazoline calix[4]arenes.

The synthesis of the *tert*-butyloxazoline calix[4]arenes **4a**/**4b** from carboxyl calix[4]arenes **3a**/**3b** was achieved through one of two standard three-step methods (Scheme 2). These calix[4]arenes were then subjected to ortholithiation conditions varying the solvent, alkyllithium and ligand. The results are reported in Table 1, which for the purposes of comparison, also includes data from our previously published work on the isopropyloxazoline calix[4]arenes **1a** and **1b** [27–28]. The conversion values and diastereomer ratios were determined either by NMR spectroscopy or HPLC traces. The assignment of the configuration of the major/minor diastereomers when using the *tert*-butyloxazoline directing group was confirmed by hydrolysis of the oxazoline and comparison with an identical sample of known configuration (see Supporting Information File 1 for full details).

**Table 1:** Results of ortholithiation study.^a^

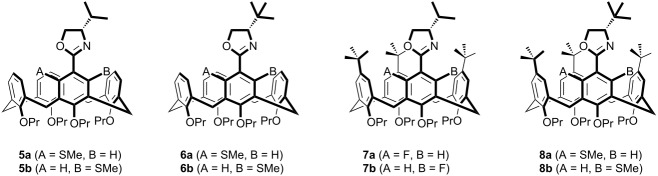						

Entry^b^	Solvent	RLi	dr **5a:5b** (conv %)	dr **6a:6b** (conv %)	dr **7a:7b** (conv %)	dr **8a:8b** (conv %)

1	Et_2_O	*n*-BuLi	1:43 (50)	1:6 (18)	–	–
2	Et_2_O	*s*-BuLi	1:13 (95)	1:8 (94)	1:7 (66)	1:380 (80)^c^
3	Et_2_O	*t*-BuLi	1:1 (70)	12:1 (75)^d^	0	69:1 (61)^c^
4	Et_2_O	iPrLi	1:30 (90)	1:62 (92)	1:11 (65)^d^	>1:200 (25)
5	Et_2_O	*c*-PentLi	1:14 (90)	1:23 (80)	1:27 (78)^d^	>1:200 (30)
6	pentane	*n*-BuLi	1:43 (47)	–	–	–
7	pentane	*s*-BuLi	1:14 (97)	1:120 (95)	1:2 (8)	1:199 (46)
8	pentane	*t*-BuLi	1:5 (95)	10:1 (50)	–	–
9	pentane	iPrLi	1:30 (95)	1:115 (67)	–	–
10	pentane	*c*-PentLi	1:35 (93)	1:57 (67)	–	–

^a^Results recorded with a ‘–‘ gave <5% conversions; ^b^general reaction conditions: (i) calix[4]arene **1a**, **1b**, **4a** or **4b**, RLi (5 equiv), TMEDA (10 equiv), −78 °C, 7 h; (ii) Me_2_S_2_ (excess), warm to rt; ^c^ortholithiation for 48 h; ^d^ortholithiation for 24 h.

Over the course of this work we have found that the lithiations are best performed at −78 °C and at a concentration of 0.15 M with either 5 or 6 equivalents of alkyllithium. Higher temperatures unsurprisingly resulted in poorer diastereoselectivity, pointing to kinetic control. A lower diastereoselectivity was also seen for higher reaction concentrations, which has been attributed to solubility and mixing problems. The excess of alkyllithium has been found to result in more convenient lithiation times (4–7 hours), although the *tert*-butyl calix[4]arenes typically required much longer lithiation times (24–48 h). In all cases the ligand *N*,*N*,*N*',*N*'-tetramethylethylenediamine (TMEDA) was crucial for success.

Our first observation, when looking at the results in Table 1, was that although the starting materials may be considered structurally similar, there was no correlation between which method would be the preferred approach to synthesising inherently chiral calix[4]arenes. Nevertheless, the hypothesis that the *tert*-butyloxazoline would allow for greater diastereoselectivity in calix[4]arenes **6** and **8** was essentially confirmed. The values obtained were far superior to those recorded with the isopropyloxazoline directed ortholithiation (see for example Table 1, entry 2, **8a**:**8b** and entry 7, **6a**:**6b**). We were however pleasantly surprised by the results when using *tert*-butyllithium in conjunction with the *tert*-butyloxazoline calix[4]arenes (Table 1, entries 3 and 8). Here the ratio of diastereomers was found to be opposite to those obtained when using the other alkyllithiums. Indeed diastereoselective reversals of this type have only rarely been observed in the literature [28–31] but importantly offer the advantage of accessing both inherently chiral antipodes after removal of the oxazoline. This avoids the need to resort to the more expensive oxazoline enantiomer derived from D-*tert-*leucine.

### Probing the impact of inherently chiral calix[4]arenes

**Figure 1:**
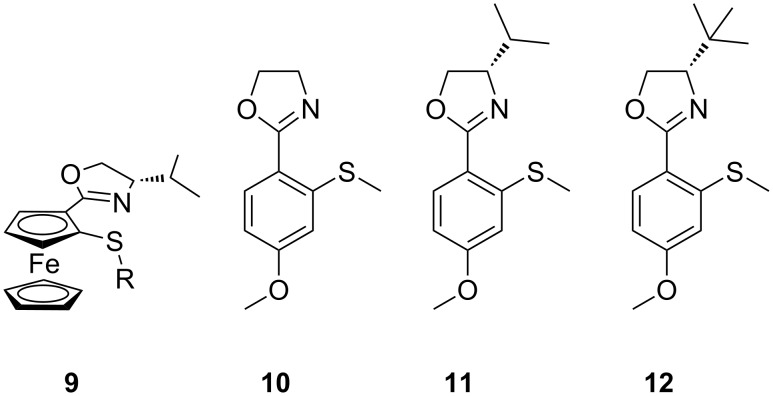
N,S Ligands.

**Scheme 3:**
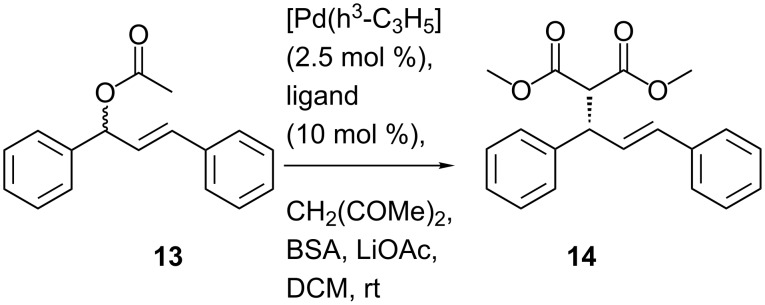
Tsuji–Trost reaction.

To date we have focused on developing the synthetic methods for *meta*-functionalised inherently chiral calix[4]arenes, our reasoning being that these are structurally related to planar chiral ferrocenes [32–34] and should thus behave in a similar fashion. To date though, we had not been in a position to examine whether this hypothesis was true. However, with the synthesis of inherently chiral thioether oxazoline calixarenes **5**–**8**, we noted their similarity to planar chiral thioether oxazoline ferrocene ligands (**9** in Figure 1) already reported by Dai and co-workers [35]. We therefore initiated a pilot study with our inherently chiral calix[4]arenes, using the palladium-catalyzed Tsuji–Trost allylation reaction (Scheme 3) as our means of probing the inherent chirality of our ligands. For comparison we also synthesised planar ligands **10**–**12** using conventional methods (see Supporting Information File 1). The catalytic runs were conducted under the identical conditions reported by Dai (for comparison purposes) and are reported in Table 2.

**Table 2:** Results of the Tsuji–Trost reaction.

Entry	Ligand	Time (h)	Yield (%)	er^a^ (*R:S*)

1	**10**	72	72	51:49
2	**11**	72	86	28:72
3	**5a**	24	93	33:67
4	**5b**	24	95	26:74
5	**12**	72	92	10:90
6	**6a**	24	98	12:88
7	**6b**	24	97	5:95
8	**7b**	1	–	
9	**8a**	1	–	
10	**8b**	1	–	

^a^Determined by chiral HPLC.

The first thing we noted was that calixarene ligands **5** and **6** showed good activity, not only in returning excellent conversions to the product, but also in reducing the reaction time compared to that of the ‘flat’ model ligands (24 h vs 72 h). This observation was also noted by Dai and co-workers with respect to their planar chiral ferrocene derivatives (e.g., **9**). Although it is unclear to us why the rate should be enhanced, the fact that it does bodes well for calix[4]arenes being investigated as ligands. On a less positive note, ligands **7b** and **8** were completely ineffective, with palladium black precipitating out of the reaction medium within 30 minutes of reaction. In order to confirm that the stability of the complex was the cause, a solution containing only the catalyst and ligand was left to stir, with decomposition of the complex occurring within a similar timeframe. We have speculated that the additional steric bulk surrounding the coordination centre is responsible for the instability of the complex, thus promoting the formation of palladium black.

When comparing the enantiomeric ratios, the results show that all four inherently chiral calix[4]arene ligands promote the formation of the (*S*)-configured product **14**. The majority of the chiral induction is clearly due to the point chirality on the oxazoline group which can be seen to increase as the steric bulk of the chiral centre increased (entries 1,2 and 5, Table 2). However, it is also clear that the inherently chiral aspect of the ligands has only a small impact on the enantiomeric ratios. In each pair of results for the calix[4]arene ligands (entries 3/4 and 6/7, Table 2), it can be seen that a small but discernible matched/mismatched result is recorded. The (*cS*) diastereomers (**6a**/**7a**) displayed a greater selectivity for the (*S*)-configured product when compared to their equivalent (*cR*) diastereomers (**6b**/**7b**). This, however, is entirely consistent with the findings of Dai et al. who also reported the same observation on their planar chiral ferrocene ligands [35].

## Conclusion

To conclude, we have reported new optimized reaction conditions toward the selective synthesis of inherently chiral *tert*-butyloxazoline calix[4]arenes (**6a**/**6b**) and *tert*-butyloxazoline *tris*(*tert*-butyl) calix[4]arenes (**8a**/**8b**). Significantly, we have shown that by merely changing the choice of alkyllithium, both the (*cS*) and (*cR*) diastereomers are accessible. Furthermore, four inherently chiral bidentate (S/N) calix[4]arene ligands were evaluated using the Tsuji–Trost allylation reaction as an asymmetric probe. The results from this catalytic study show that these ligands were both efficient and selective, but the selectivity was largely due to the point chirality of the oxazoline and not the inherent chirality of the calixarene. Since these results are entirely consistent with those of the planar chiral ferrocene system reported by Dai et al., we believe future ligand design may better exploit the inherently chiral calix[4]arene. Currently we are looking at different catalytic systems, also based on the planar chiral ferrocenes, but which have shown the planar chirality of the ferrocene to be essential in determining the selectivity and/or efficiency of the transformations. Our ultimate aim is to establish whether or not the inherent chirality on the calix[4]arenes can be also be used to drive asymmetric transformations/reactions.

## Experimental

See Supporting Information File 1.

## Supporting Information

Experimental details and characterization data for all products; determination of enantiomeric ratios and determination of major calix[4]arene diastereomer.

File 1 Synthetic procedures and spectral data for all new compounds.
