# Correction: Inherently chiral calix[4]arenes via oxazoline directed ortholithiation: synthesis and probe of chiral space

**DOI:** 10.3762/bjoc.13.38

**Published:** 2017-02-23

**Authors:** Simon A Herbert, Laura J van Laeren, Dominic C Castell, Gareth E Arnott

**Affiliations:** 1 Department of Chemistry and Polymer Science, Stellenbosch University, Private Bag X1, Matieland 7602, South Africa

**Keywords:** calix[4]arene, inherently chiral, ortholithiation, oxazoline, Tsuji–Trost

In Supporting Information File 1 of the original article a wrong flow rate and wrong retention times were reported on page s29. The correct flow rate is 1.1 mL/min and the correct retention times are Rt_1_: 6.01 (R) and Rt_2_: 6.64 (S). A corrected version of the Supporting Information File of the original article is given as Supporting Information File 1 of this Correction.

## Supporting Information

File 1 Synthetic procedures and spectral data for all new compounds.

