# Supplementary material for: Inherently chiral calix[4]arenes via oxazoline directed ortholithiation: synthesis and probe of chiral space
Source: Beilstein J Org Chem. 2014 Nov 25;10:2751–5. doi: 10.3762/bjoc.10.291 (PMC4273304; doi:10.3762/bjoc.10.291)
Supplement: File 1 — Synthetic procedures and spectral data for all new compounds. [file Beilstein_J_Org_Chem-10-2751-s001.pdf]

**Supporting Information**

**for**

# Inherently chiral calix[4]arenes via oxazoline directed ortholithiation: synthesis and probe of chiral space


Simon A. Herbert[1], Laura J. van Laeren[1], Dominic C. Castell[1] and Gareth E. Arnott*[1]

Address: [1]Department of Chemistry and Polymer Science, Stellenbosch University, Private Bag X1, Matieland 7602, South Africa,

Email: Gareth Arnott - arnott@sun.ac.za

* Corresponding author


**Synthetic procedures and spectral data for all new compounds.**

# Contents

1. **General practices** .................................................................................................. s3

2. **Synthesis of inherently chiral calixarenes** ....................................................................... s6

3. **Synthesis of 'flat' model ligand compounds.** ....................................................................... s24

4. **Asymmetric catalytic study: Synthesis of materials and results.** .................................... s28

5. **Identification of the major/minor diastereomers for the tBuOx calixarene** .................. s32

6. **References** .................................................................................................................. s34

## 1. General practices

### *Solvents and reagents*

Chemicals used in these experiments were purchased from Merck or Aldrich. Tetrahydrofuran, diethyl ether, toluene and pentane were distilled under nitrogen from sodium wire/sand using benzophenone as an indicator; alternative they were dried in a sealed Schlenk flask with 20% molecular sieves. Solvent was also collected under inert conditions from an Innovative Technologies PureSolve PS-MP-5 solvent purification system. Dichloromethane was distilled under nitrogen from calcium hydride. Other reagents requiring purification were purified according to standard procedures.

### *Temperature control*

Low temperature reactions were performed in a Dewar containing dry ice in acetone (–78 °C), or a slurry of ethanol, sodium chloride and ice (–20 °C). Reactions requiring precise, extended low temperature control, were performed in a Dewar regulated with a Thermo Scientific Haake EK90 Immersion Cooler. Microwave reactions were performed in a Biotage Initiator microwave reactor.

### *Inert conditions*

Glassware was dried for at least an hour at 120 °C, thereafter it was placed under vacuum of <0.5 mm Hg and cyclically flushed with nitrogen/argon and evacuated until it had reached RT. Standard Schlenk techniques were employed when necessary. All reactions were performed under a positive pressure of 2.8 kPa of 5.0 grade nitrogen or argon (Air Products).

### *Chromatography*

All column chromatography was performed on Merck silica gel 60 (particle size 0.040-0.063 mm) using one of or combinations of petroleum ether, diethyl ether, ethyl acetate, toluene, dichloromethane, ethanol and methanol as a solvent. Thin layer chromatography (TLC) was carried out on aluminium backed Merck silica

gel 60 $F_{254}$ plates. Visualization was performed with a UV lamp, using iodine on silica, or by spraying with a Cerium Ammonium Molybdate (CAM) or ninhydrin (NIN) solution followed by heating.

### *Characterization*

All $^{1}H$, $^{13}C$, $^{19}F$ and $^{31}P$ nuclear magnetic resonance spectra were obtained using a 300 MHz Varian VNMRS (75 MHz for $^{13}C$), 400 MHz Varian Unity Inova (100 MHz for $^{13}C$), 600 MHz Varian Unity Inova (150 MHz for $^{13}C$) with deuterated solvents. Chemical shifts (δ) were recorded using the residual solvent peak or external reference. All chemical shifts are reported in ppm and all spectra were obtained at 25 °C unless otherwise reported. Data was processed using MestReNova.

Melting points were obtained using a Gallenkamp Melting Point Apparatus or a Kofler microscope melting point machine and are uncorrected. Infrared spectra were obtained using a Nexus Thermo-Nicolet FT-IR instrument using thin film (NaCl plate), or using the ATR attachment or using an ATi Perkin Elmer Spectrum RX1 FTIR spectrometer using thin film (NaCl). High resolution mass spectrometry was performed by CAF (Central Analytical Facility) at Stellenbosch University using a Waters API Q-TOF Ultima spectrometer, and at the University of Manchester on a Thermo Finnigan MAT 95XP mass spectrometer, or at the EPSRC National Centre Swansea using a Thermofisher LTQ Orbitrap XL. Routine mass spectroscopy was done using a Waters API Quattro Micro spectrometer at Stellenbosch University or at the University of Manchester using a Micromass Platform II. Optical rotations were recorded using a Bellingham + Stanley ADP 220 Polarimeter using a 1 dm cell or using Perkin-Elmer 241 Polarimeter using a cell with a path length of 0.25 dm.

### *Previously reported compounds*

Compounds **1a**, **5a** and **5b**[1] and compounds **1b** and **7b**[2] have reported before and were synthesized as per the literature procedures.

### *General ortholithiation procedure*

The alkyllithium (5 eq) was added to an oven dried Schlenk flask under argon at RT. The flask was cooled (0 °C) and the excess solvent was removed under reduced pressure. Dry solvent (reactions were typically

0.15 M) was added to the alkyllithium and the resulting mixture was cooled (–78 °C). TMEDA/ligand (10 eq) was then carefully added and the mixture was stirred for 15 min. Adding the TMEDA/ligand usually resulted in a colour change, from white to either orange or yellow, as well as the formation of a precipitate. The oxazoline calixarene was added to dry solvent, which was then carefully added, drop wise, to the cooled mixture. After stirring for the chosen reaction time, dimethyldisulfide (exs) was added, resulting in the disappearance of the colour and/or precipitate. The reaction mixture was then allowed to warm to RT overnight, and was finally quenched with $H_2O$. The crude product was extracted using EtOAc, washed with $H_2O$ and dried over $MgSO_4$. The excess solvent was then removed under reduced pressure. The thioether calixarene ligands were then purified using silica gel column chromatography.

## 2. Synthesis of inherently chiral calixarenes

**5-((*S*)-4-*tert*-Butyl-4,5-dihydrooxazol-2-yl)-25,26,27,28-tetrapropoxycalix[4]arene. (4a)**

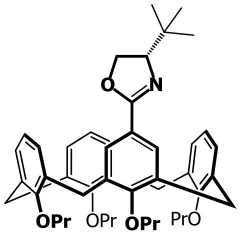


Carboxyl calixarene (**3a**) (230 mg, 0.36 mmol), oxalyl chloride (0.1 ml, 1.1 mmol, 3 eq) and DCM (2.5 ml) were added to an oven dried flask and stirred at RT for 3 hours. The solvent was removed under reduced pressure and the resulting solid was re-suspended in dry DCM (3 ml) and slowly added to a mixture of L-*tert*-leucinol (50 mg, 0.43 mmol, 1.2 eq), $Et_3N$ (0.3 ml, 2.2 mmol, 6 eq) in DCM (3 ml) at 0 °C and stirred overnight. Mesyl chloride (0.1 ml, 1.1 mmol, 3 eq) was slowly added and the flask stirred for a further 3 hours, following which $H_2O$ (5 ml) and DCM (20 ml) were added, the layers separated and the aqueous layer washed with DCM (15 ml). The organic phases were combined, dried over $MgSO_4$ and the solvent removed under reduced pressure with purification via silica gel column chromatography (EtOAc: PET, 10:90) affording a white glass (206 mg, 0.29 mmol, 80% yield). Mp 144 – 146 °C (THF); $[\alpha]_D^{18.4}$ = –16.3 (c 2.2, DCM); $R_f$ = 0.52 (EtOAc: PET, 10:90); IR (ATR): 2961 (m, –C–H stretch), 1653 (s, C=N stretch), 1557 (m, C=C stretch), 1455 (s, C=C stretch), 1193 (m, C–O stretch), 1006 (m, C–O stretch), 967 (m, C–H oop bend), 758 (s, C–H oop bend); $^1$H NMR (300 MHz, CHLOROFORM-*d*) 0.90 - 0.99 (m, 15 H, $C(CH_3)_3$, $CH_2CH_3$), 1.06 (t, *J* = 7.4 Hz, 6 H, $CH_2CH_3$), 1.82 - 2.03 (m, 8 H, $CH_2CH_2CH_3$), 3.12 - 3.26 (m, 4 H, $ArCH_2Ar$), 3.76 (t, *J* = 7.0 Hz, 4 H, $OCH_2CH_2$), 3.91 - 4.07 (m, 5 H, $OCH_2CH_2$, $OCH_2CHN$), 4.21 (dd, *J* = 7.7, 8.6 Hz, 1 H, $OCH_2CHN$), 4.32 (dd, *J* = 8.6, 10 Hz, 1 H, $OCH_2CHN$), 4.46 (d, *J* = 13.4 Hz, 4 H, $ArCH_2Ar$), 6.31 - 6.43 (m, 6 H, Ar*H*), 6.72 (m, *J* = 9.7, 1.2 Hz, 1 H, Ar*H*), 6.84 - 6.89 (m, 2 H, Ar*H*), 7.48 (d, *J* = 2.2 Hz, 1 H, Ar*H*), 7.54 (d, *J* = 2.1 Hz, 1 H, Ar*H*) δ ppm; $^{13}$C NMR (75 MHz, CHLOROFORM-*d*) 10.0, 10.6, 23.0, 23.1, 23.4, 25.9, 30.8, 30.8, 30.9, 30.9, 34.0, 68.5, 76.1, 76.5, 76.6, 76.7, 76.8, 121.2, 121.7, 122.0, 127.7, 127.8, 128.5, 128.6, 128.7, 133.4, 133.5, 134.1, 134.1, 136.1, 136.2, 136.2, 155.7, 157.3, 160.2, 163.5 δ ppm; HRMS– TOF MS ES+: m/z $[M+H]^+$ calcd for $C_{47}H_{60}NO_5$:718.4471; found: 718.4460.

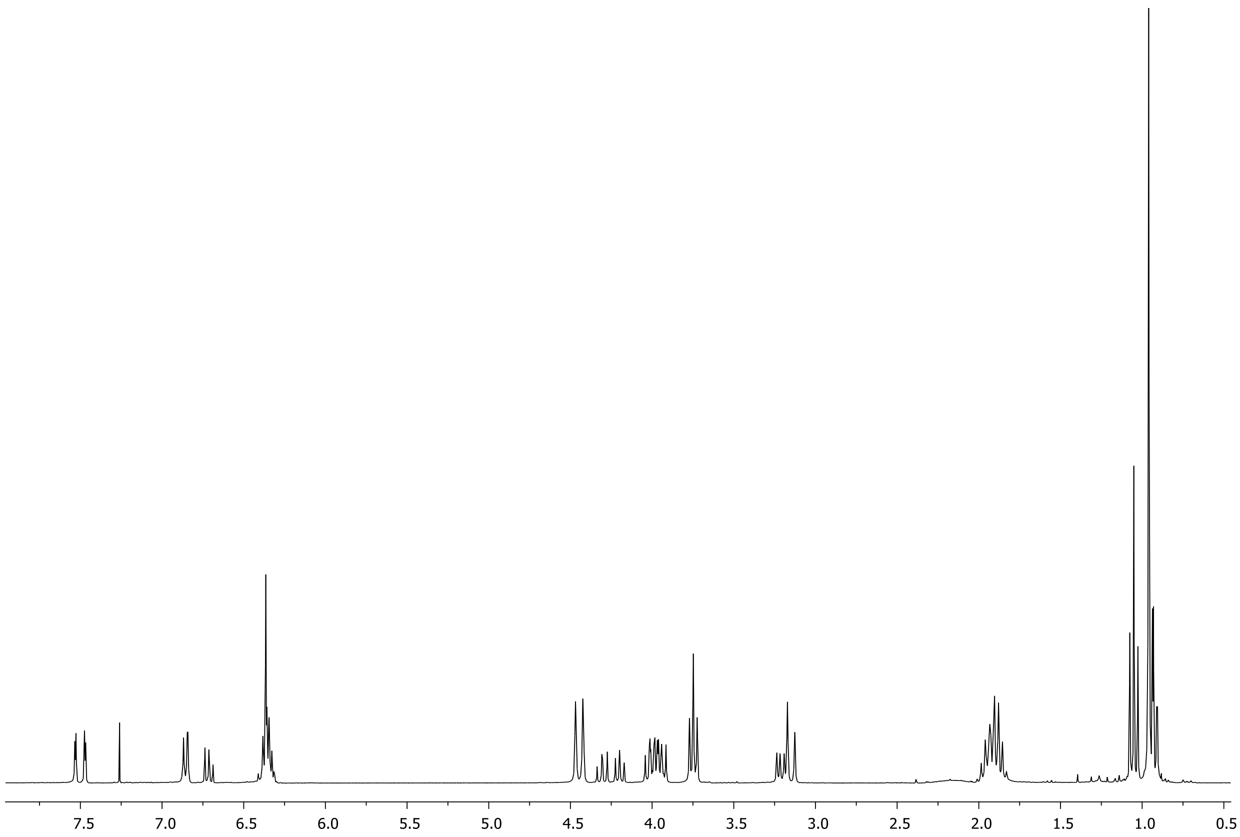


**Figure 1 - $^{1}$H NMR: 5-((*S*)-4-*tert*-Butyl-4,5-dihydrooxazol-2-yl)-25,26,27,28-tetrapropoxycalix[4]arene.**

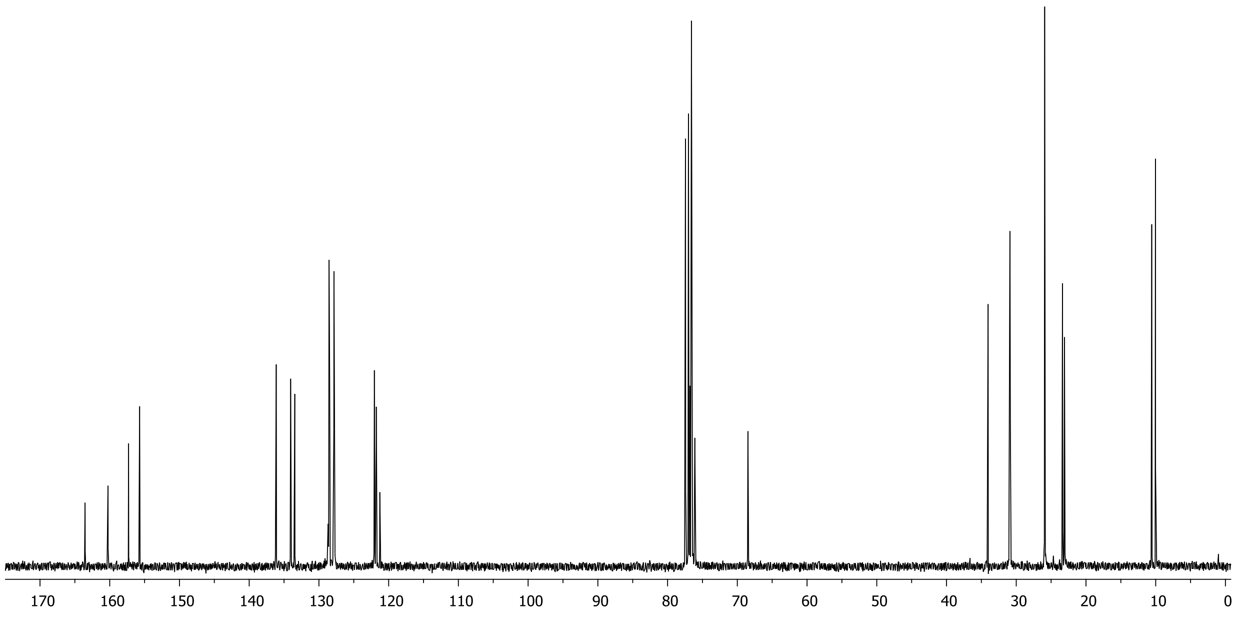


**Figure 2 – $^{13}$C NMR: 5-((*S*)-4-*tert*-Butyl-4,5-dihydrooxazol-2-yl)-25,26,27,28-tetrapropoxycalix[4]arene.**

**(*cR*)-5-((*S*)-4-*tert*-Butyl-4,5-dihydrooxazol-2-yl)-4-methylthio-25,26,27,28-tetrapropoxycalix[4]arene – (6b) (major)**

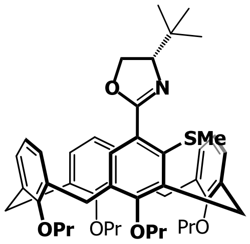

Thioether (6b) was synthesised according to the general procedure using oxazoline (4a) (20 mg, 0.03 mmol), *s*BuLi (0.14 mmol, 5 eq), TMEDA (0.04 ml, 0.28 mmol, 10 eq), $Et_2O$ (0.5 ml) and dimethyl disulphide (1 mmol, exs). Mp 94 – 139 °C (foam); $R_f$ = 0.52 (EtOAc: PET, 10:90); $[\alpha]_D^{25.0}$ = –3.5 (c 1.0, DCM); IR (ATR): 2959 (s, –C–H stretch), 1652 (s, C=N stretch), 1456 (s, C=C stretch), 1195 (m, C–O stretch), 967 (m, C–H oop bend), 758 (s, C–H oop bend); $^1$H NMR (400 MHz, CHLOROFORM-d) 0.89 (m, 6 H, $CH_2C\mathit{H_3}$), 1.06 (s, 9 H, $C(C\mathit{H_3})_3$), 1.10 (m, 6 H, $CH_2C\mathit{H_3}$), 1.81 - 2.02 (m, 8 H, $CH_2C\mathit{H_2}CH_3$), 2.42 (s, 3 H, $SC\mathit{H_3}$), 3.12 - 3.22 (m, 3 H, $ArC\mathit{H_2}Ar$), 3.62 - 3.78 (m, 5 H, $ArC\mathit{H_2}Ar$, $OC\mathit{H_2}CH_2$), 4.01 (m, 4 H, $OC\mathit{H_2}CH_2$), 4.13 (dd, *J* = 10.2, 8.2 Hz, 1 H, $OCH_2CHN$), 4.29 - 4.35 (m, 2 H, $OCH_2CHN$), 4.39 - 4.50 (m, 4 H, $ArC\mathit{H_2}Ar$), 5.97 (dd, *J* = 7.4, 1.4 Hz, 1 H, Ar*H*), 6.06 (dd, *J* = 7.7, 1.4 Hz, 1 H, Ar*H*), 6.10 - 6.19 (m, 3 H, Ar*H*), 6.24 (t, *J* = 7.6, 1 H, Ar*H*), 6.92 (t, *J* = 7.6, 1 H, Ar*H*), 7.11 (d, *J* = 7.4 Hz, 2 H, Ar*H*), 7.34 (s, 1 H, Ar*H*) δ ppm; $^{13}$C NMR (75 MHz, CHLOROFORM-*d*) 9.96, 9.97, 10.9 , 21.5, 23.12, 23.14, 23.6, 26.3, 27.4, 29.9, 30.9, 31.1, 31.2, 34.2, 69.2, 76.6, 76.82, 76.83, 77.1, 121.9, 122.3, 122.4, 127.2, 127.4, 127.7, 127.8, 129.0, 129.1, 130.1, 132.2, 132.9, 133.2, 133.4, 133.6, 137.2, 137.3, 137.7, 142.5, 143.2, 155.15, 155.21, 158.2, 160.4, 165.5 *δ* ppm; HRMS– TOF MS ES+: m/z $[M+H]^+$ calcd for $C_{48}H_{62}NO_5S$:764.4349; found: 764.4357.

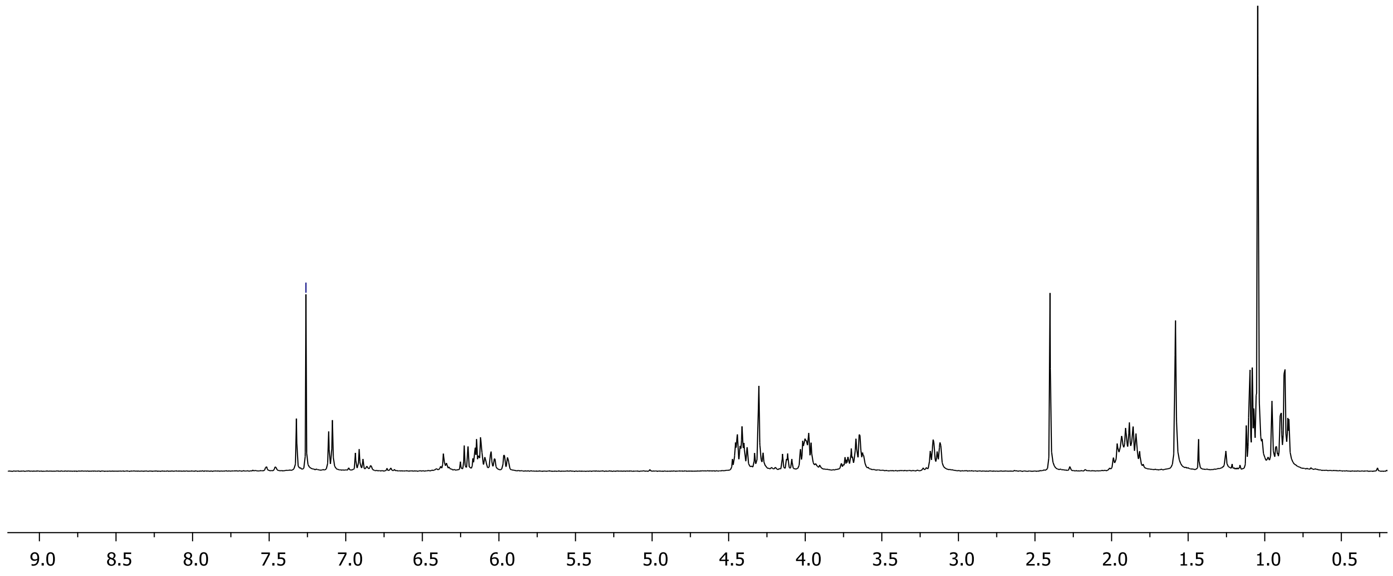


**Figure 3 – $^{1}$H NMR: (*cR*)-5-((*S*)-4-*tert*-Butyl- 4,5-dihydrooxazol-2-yl)-4-methylthio-25,26,27,28-tetrapropoxycalix[4]arene**

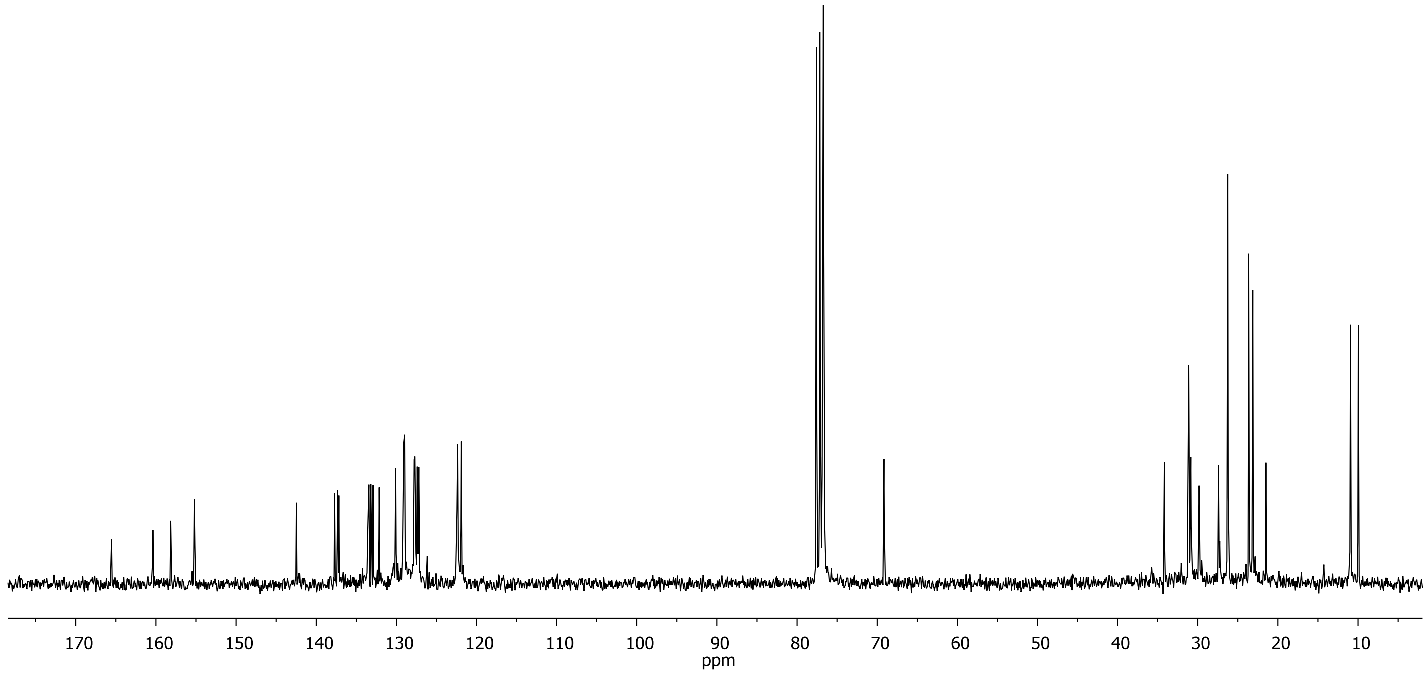


**Figure 4 - $^{13}$C NMR: (*cR*)-5-((*S*)-4-*tert*-Butyl-4,5-dihydrooxazol-2-yl)-4-methylthio-25,26,27,28-tetrapropoxycalix[4]arene**

**(*c*S)-5-((*S*)-4-*tert*-Butyl-4,5-dihydrooxazol-2-yl)-4-methylthio-25,26,27,28-tetrapropoxycalix[4]arene – (6a)**

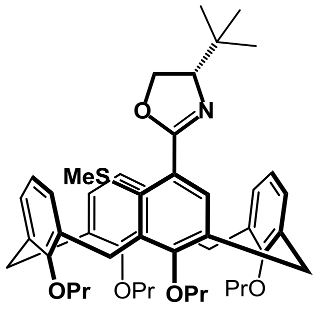


Thioether **(6a)** was synthesised according to the general ortholithiation procedure using oxazoline **(4a)** (20 mg, 0.03 mmol), *s*BuLi (0.14 mmol, 5 eq), TMEDA (0.04 ml, 0.28 mmol, 10 eq), $Et_2O$ (0.5 ml) and dimethyl disulphide (1 mmol, exs). Mp 94 – 139 °C (foam); $R_f$ = 0.52 (EtOAc: PET, 10:90); $[\alpha]_D^{25.0}$ = –3.5 (c 1.0, DCM); IR (ATR): 2959 (s, –C–H stretch), 1652 (s, C=N stretch), 1456 (s, C=C stretch), 1195 (m, C–O stretch), 967 (m, C–H oop bend), 758 (s, C–H oop bend); $^1$H NMR (300 MHz, CHLOROFORM-*d*) 0.86 (t, *J* = 7.5 Hz, 3 H, $CH_2CH_3$), 0.88 (t, *J* = 7.5 Hz, 3 H, $CH_2CH_3$), 1.05 (s, 9 H, $C(CH_3)_3$), 1.08 (t, *J* = 7.4 Hz, 3 H, $CH_2CH_3$), 1.10 (t, *J* = 7.4 Hz, 3 H, $CH_2CH_3$), 1.82 – 1.98 (m, 8 H, $CH_2CH_3$), 2.41 (s, 3 H, $SCH_3$), 3.12 – 3.18 (m, 3 H, $ArCH_2Ar$), 3.62 – 3.74 (m, 5 H, $ArCH_2Ar$, $OCH_2CH_2$), 3.96 – 4.03 (m, 4 H, $OCH_2CH_2$), 4.12 (dd, *J* = 9.7, 7.8 Hz, 1 H, $OCH_2CHN$), 4.25 – 4.31 (m, 2 H, $OCH_2CHN$), 4.38 – 4.46 (m, 4 H, $ArCH_2Ar$), 5.96 (dd, *J* = 7.5, 1.7 Hz, 1 H, Ar*H*), 6.05 (dd, *J* = 7.6, 1.3 Hz, 1 H, Ar*H*), 6.10 – 6.17 (m, 3 H, Ar*H*), 6.23 (t, *J* = 7.5 Hz, 1 H, Ar*H*), 6.91 (t, *J* = 7.5 Hz, 1 H, Ar*H*), 7.09 (d, *J* = 7.4 Hz, 2 H, Ar*H*), 7.29 (s, 1 H, Ar*H*) δ ppm. $^{13}$C NMR (75 MHz, CHLOROFORM-*d*) 9.9, 10.0, 10.9, 10.9, 21.6, 23.1, 23.2, 23.7, 26.3, 27.8, 29.9, 30.9, 31.1, 31.2, 34.2, 69.0, 76.7, 76.8, 76.9, 77.1, 77.4, 77.7, 121.9, 122.3, 122.4, 127.3, 127.4, 127.7, 127.8, 128.9, 129.0, 129.1, 130.0, 132.3, 133.1, 133.3, 133.5, 133.7, 137.1, 137.3, 137.7, 142.5, 155.2, 155.3, 158.2, 160.3, 165.2 δ ppm. HRMS– TOF MS ES+: m/z $[M+H]^+$ calcd for $C_{48}H_{62}NO_5S$:764.4349; found: 764.4343

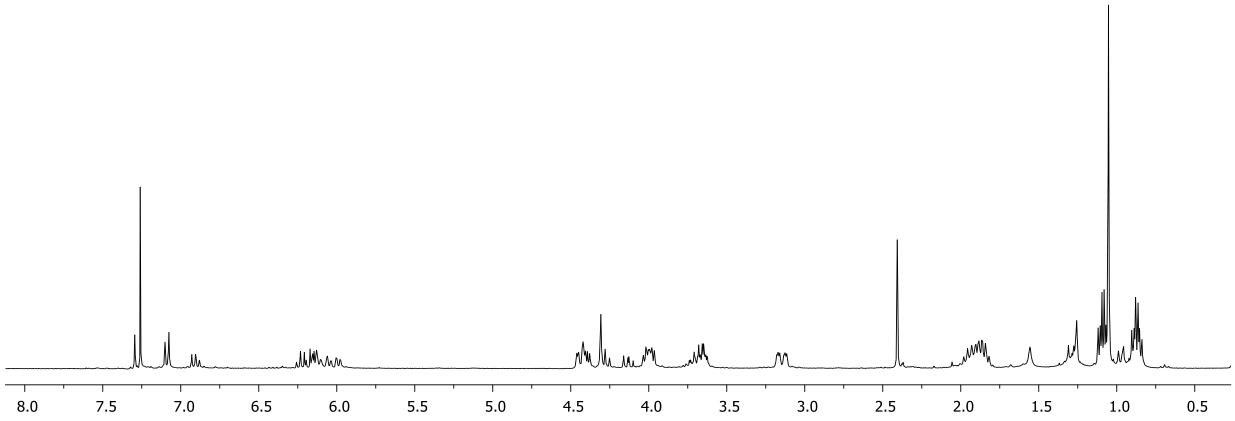


**Figure 5 – $^{1}$H NMR: (*cS*)-5-((*S*)-4-*tert*-Butyl-4,5-dihydrooxazol-2-yl)-4-methylthio-25,26,27,28-tetrapropoxycalix[4]arene**

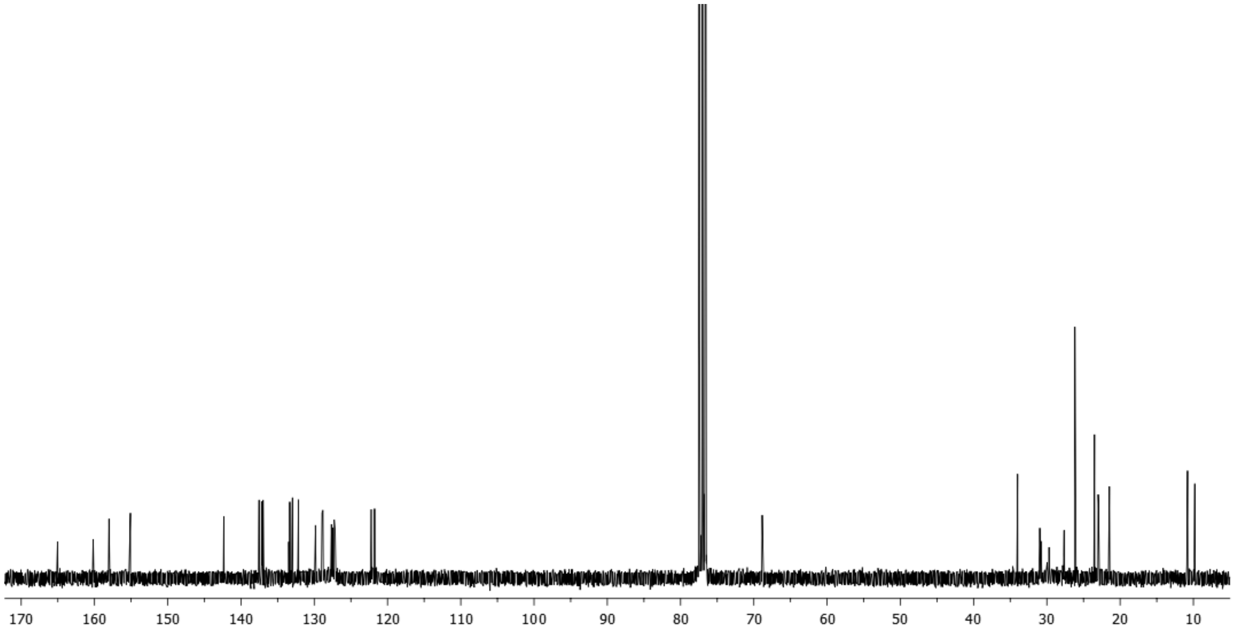


**Figure 6 - $^{13}$C NMR: (*cS*)-5-((*S*)-4-*tert*-Butyl-4,5-dihydrooxazol-2-yl)-4-methylthio-25,26,27,28-tetrapropoxycalix[4]arene**

**11,17,23-Tri-*tert*-butyl-5-((*S*)-4-*tert*-butyl-4,5-dihydrooxazol-2-yl)-25,26,27,28-tetrapropoxycalix[4]arene (4b)**

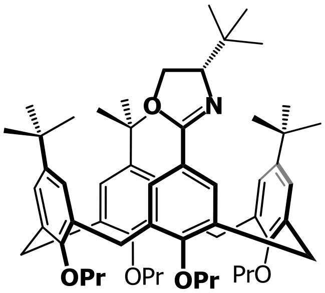


Carboxyl calixarene **(3b)** (552 mg, 0.687 mmol) and thionyl chloride (3.0 ml, 41 mmol, 60 eq) were added to an oven dried bottom flask (50 ml) fitted with a reflux condenser. The solution was stirred under argon at RT for 10 minutes, after which it was brought to reflux for 2 hours. The excess thionyl chloride was removed under reduced pressure while maintaining inert conditions, affording an orange foam. The solid was re-dissolved in dry DCM (5.0 ml) and slowly added to a mixture of L-*tert*-leucinol (100.0 mg, 0.82 mml, 1.2 eq), $Et_3N$ (0.34 ml, 2.5 mmol, 3.6 eq) in DCM (15.0 ml) at 0 °C. The mixture was allowed to warm to RT and stirred overnight. The contents of the flask was taken up in additional DCM (50 ml) and washed with sodium bicarbonate solution (20 ml). The aqueous layer was extracted with DCM (3 × 100 ml), the organic layers were combined, dried over $MgSO_4$ and the solvent was removed under reduced pressure yielding a light yellow solid. The solid was dried under high vacuum following which it was re-dissolved in dry DCM (15.0 ml) and transferred to a dry reaction vessel under argon. Thionyl chloride (0.8 ml, 11.3 mmol, 16.4 eq) was added to the mixture which was stirred for 12h at RT. Water was then carefully added to the mixture until frothing ceased. Additional DCM (50 ml) was added to the mixture and the organic layer was washed with sat. sodium bicarbonate solution (30 ml). The aqueous layer was again extracted with 3 × 100 ml DCM. The organic layers were combined, dried over $MgSO_4$ and the excess solvent was removed under reduced pressure. Purification was achieved by silica gel column chromatography (EtOAc:PET, 2:98) yielding a white solid (571 mg, 0.64 mmol, 94 % Yield). Further purification was achieved by slow recrystallization from EtOH and DCM. Mp 209 – 210°C (EtOH/DCM); $R_f$ = 0.83 (EtOAc:PET, 10:90); $[\alpha]_D^{24.5}$ = –10.4 (c 2.2, DCM); IR (ATR) $cm^{-1}$: 2957 (m, –C–H stretch), 1654 (s, C=N stretch), 1480 (s, C=C stretch), 1201 (m, C–O stretch), 1008 (m, C–O stretch), 960 (m, C–H oop bend); $^1H$

NMR (300 MHz, CHLOROFORM-*d*) 0.77 (s, 9 H, $C(CH_3)_3$), 0.85 (s, 9 H, $C(CH_3)_3$), 0.95 (t, *J* = 7.4 Hz, 6 H, $CH_2CH_3$), 1.03 – 1.12 (m, 6 H, $CH_2CH_3$), 1.30 (s, 9 H, $C(CH_3)_3$), 1.30 (s, 9 H, $C(CH_3)_3$), 1.86 – 1.99 (m, 4 H, $CH_2CH_2CH_3$),1.99 – 2.14 (m, 4 H, $CH_2CH_2CH_3$), 3.09 – 3.20 (m, 4 H, $ArCH_2Ar$), 3.68 – 3.75 (m, 4 H, $OCH_2CH_2$), 3.76 – 3.82 (m, 1 H, $NCHCH_2$), 3.89 – 4.01 (m, 5 H, $OCH_2CH_2$, $OCH_2CH$), 4.11 (dd, *J* = 9.9, 8.5 Hz, 1 H, $OCH_2CH$), 4.40 – 4.46 (m, 4 H, ArC*H*2Ar), 6.34 (d, *J* = 2.5 Hz, 1 H, Ar*H*), 6.37 (d, *J* = 2.5 Hz, 1 H, Ar*H*), 7.02 – 7.07 (m, 3 H, Ar*H*), 7.07 – 7.11 (m, 2 H, Ar*H*), 7.13 (d, *J* = 2.0 Hz, 1 H, Ar*H*) δ ppm; $^{13}C$ NMR (75 MHz, CHLOROFORM-*d*) 10.0, 10.6, 10.7, 23.1, 23.2, 23.3, 23.5, 26.0, 30.7, 30.8, 30.9, 31.0, 31.1, 31.6, 33.4, 33.6, 34.0, 68.2, 76.1, 76.6, 76.7, 77.1, 77.2, 121.8, 124.3, 125.0, 125.4, 125.5, 125.7, 125.8, 127.7, 127.9, 132.3, 132.5, 133.6, 133.7, 134.5, 134.7, 135.4, 135.5, 143.9, 144.6, 144.7, 152.9, 154.6, 154.6, 157.9, 163.2 *δ* ppm; MS (ESI+): *m/z* (%) = 886.6 (100); [M+H]+; HRMS–TOF MS ESI+: *m/z* [M+H]+ calcd for $C_{59}H_{84}NO_5$: 886.6349; found: 886.6337.

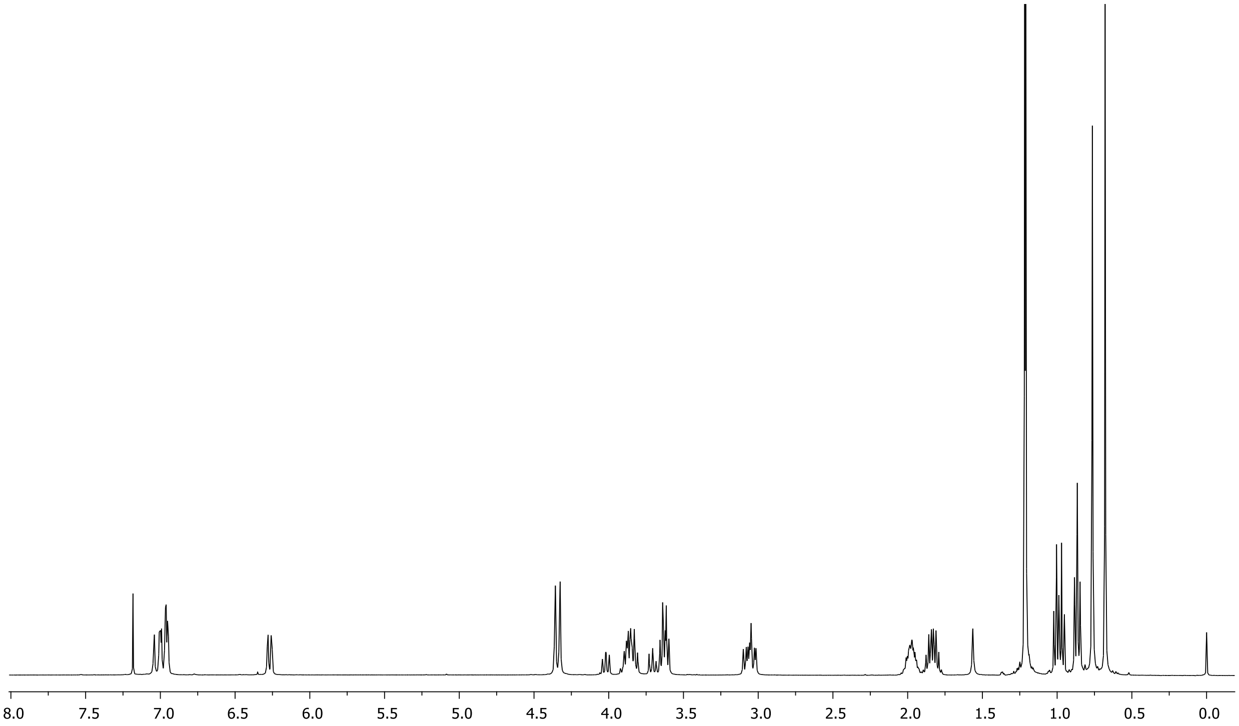


**Figure 7 – $^{1}$H NMR: 11,17,23-Tri-*tert*-butyl-5-((*S*)-4-*tert*-butyl-4,5-dihydrooxazol-2-yl)-25,26,27,28-tetrapropoxycalix[4]arene**

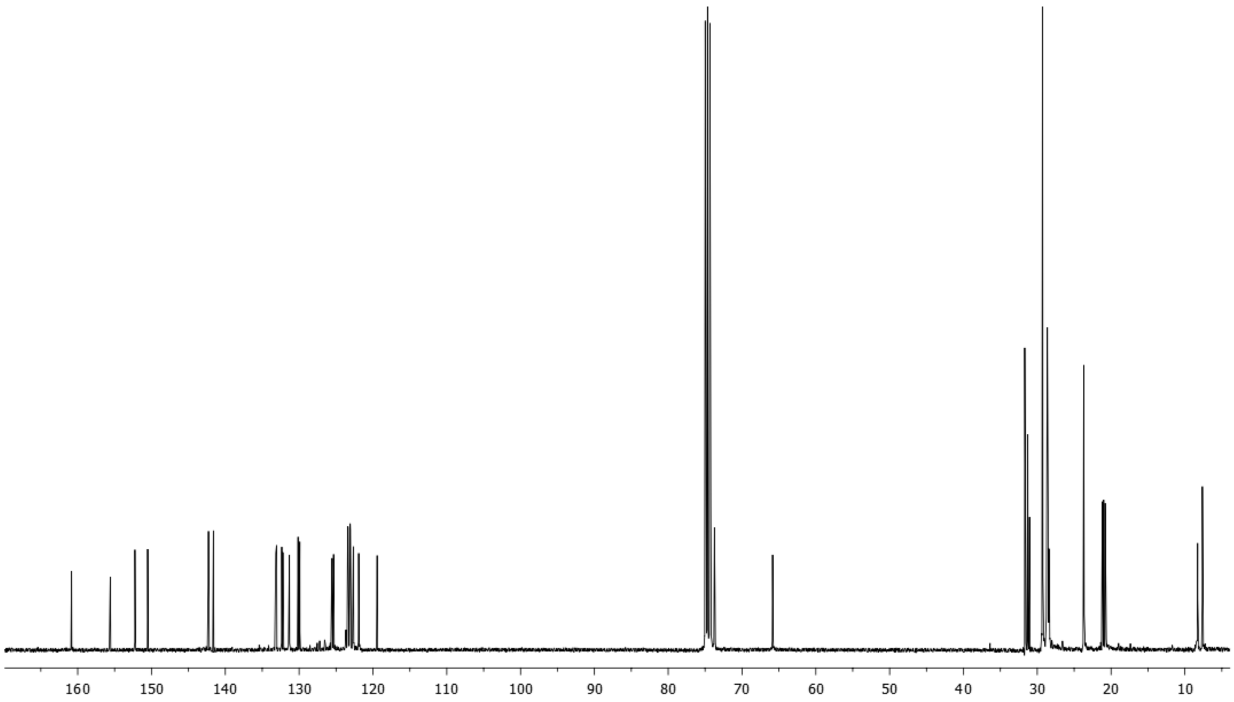


**Figure 8 – $^{13}$C NMR: 11,17,23-Tri-*tert*-butyl-5-((*S*)-4-*tert*-butyl-4,5-dihydrooxazol-2-yl)-25,26,27,28-tetrapropoxycalix[4]arene**

**(*cR*)-11,17,23-Tri-*tert*-butyl-5-((*S*)-4-*tert*-butyl-4,5-dihydrooxazol-2-yl)-4-methylthio-25,26,27,28-tetrapropoxycalix[4]arene – (8b)**

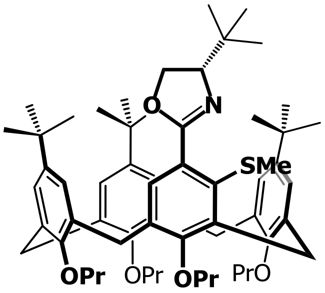


Thioether (8b) was synthesised according to the general ortholithiation procedure from oxazoline (4b) (30 mg, 0.034 mmol), *s*BuLi (0.17 mmol, 5 eq), TMEDA (0.34 mmol, 10 eq), $Et_2O$ (0.2 ml) and dimethyldisulfide (0.05 ml, 5 mmol, exs) with a reaction time of 72 hours. Yield by HPLC 57 %; $R_f$ = 0.41 (EtOAc:PET, 10:90); $[\alpha]_D^{24.5}$ = –8.4 (c 1.0, DCM); IR (film): 2959 (s, –C–H stretch), 1661 (s, C=N stretch), 1463 (s, C=C stretch), 1199 (m, C–O stretch), 968 (m, C–H oop bend), 870 (s, C–H oop bend), 800 (s, C–H oop bend) 738 (s, C–H oop bend); $^1$H NMR (500 MHz, CHLOROFORM-d) ▯ ppm 0.94 - 1.03 (m, 30 H, C(C$H_3$)$_3$, CH$_2$C$H_3$), 1.04 (s, 9 H, C(C$H_3$)$_3$), 1.13 (s, 9 H, C(C$H_3$)$_3$), 1.87 - 2.10 (m, 11 H, SC$H_3$, CH$_2$C$H_2$CH$_3$), 3.15 (d, *J* = 12.6, 2 H, ArC$H_2$Ar), 3.16 (d, *J* = 12.6, 1 H, ArC$H_2$Ar), 3.69 - 3.75 (m, 1 H, OC$H_2$CH$_2$), 3.76 - 3.94 (m, 7 H, OC$H_2$CH$_2$), 4.00 - 4.06 (m, 1 H, OCH$_2$CHN), 4.10 - 4.18 (m, 2 H, OCH$_2$CHN, ArC$H_2$Ar), 4.27 - 4.32 (m, 1 H, OCH$_2$CHN), 4.34 (d, *J* = 12.9 Hz, 1 H, OCH$_2$CHN), 4.39 - 4.42 (d, *J* = 12.6 Hz, 1 H, ArC$H_2$Ar), 4.44 (d, *J* = 12.6 Hz, 2 H, ArC$H_2$Ar), 6.67 (br. s., 2 H, Ar*H*), 6.75 (s, 1 H, Ar*H*), 6.77 (br. s., 1 H, Ar*H*), 6.84 (br. s., 1 H, Ar*H*), 6.86 (s, 1 H, Ar*H*), 7.16 (s, 1 H, Ar*H*) $\delta$ ppm; $^{13}$C NMR (75 MHz, CHLOROFORM-*d*) 10.1, 10.3, 10.4, 10.5, 21.5, 22.9, 23.3, 23.4, 26.1, 28.6, 28.7, 29.7, 30.5, 31.2, 31.4, 31.4, 31.5, 33.7, 33.8, 33.8, 33.9, 68.5, 76.6, 76.7, 76.8, 77.2, 124.6, 124.7, 125.0, 125.2, 125.3, 125.8, 125.8, 129.8, 130.0, 132.0, 132.9, 133.1, 133.4, 134.1, 134.5, 136.0, 140.8, 143.8, 144.3, 144.4, 153.6, 153.7, 154.1, 158.7, 165.0 ▯ ppm; MS (ESI+): *m/z* (%) = 932 (100) [M+H]$^+$; HRMS– TOF MS ESI+: *m/z* [M+H]$^+$ calcd for $C_{60}H_{86}NO_5S$: 932.6222; found: 932.6235.

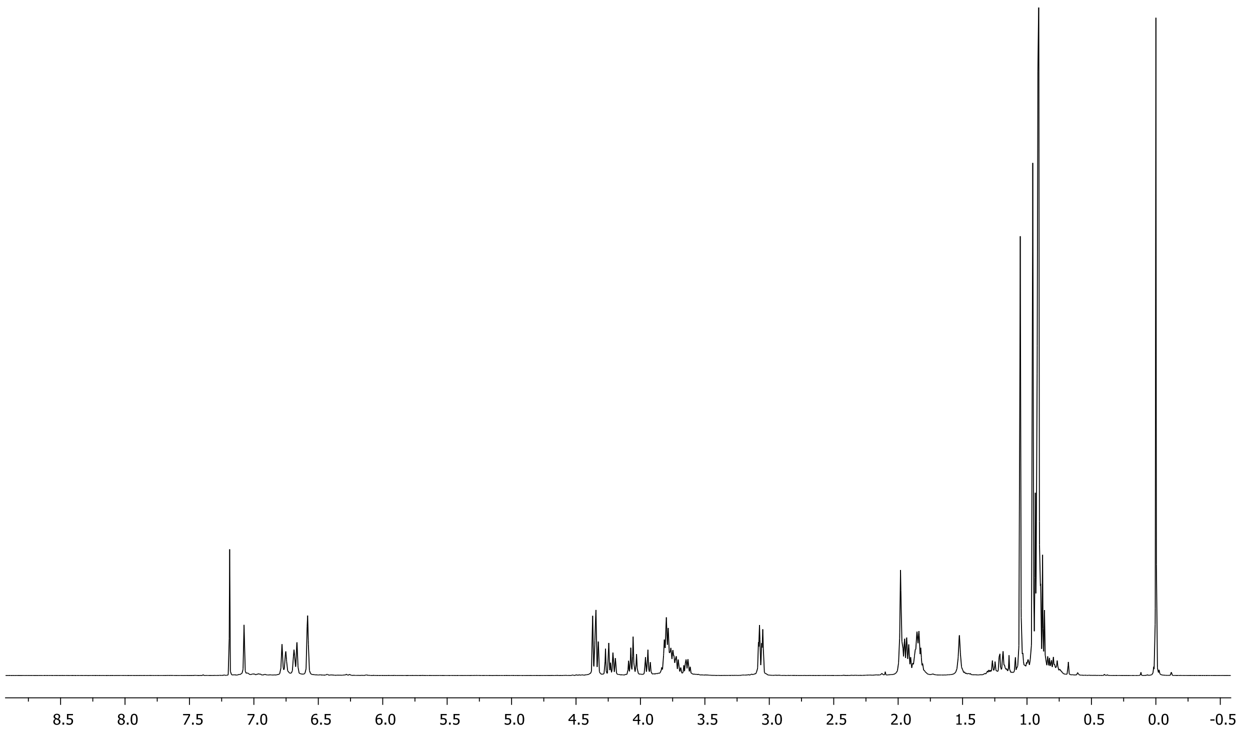


**Figure 9 – $^{1}$H NMR: (*cR*)-11,17,23-Tri-*tert*-butyl-5-((*S*)-4-*tert*-butyl-4,5-dihydrooxazol-2-yl)-4-methylthio-25,26,27,28-tetrapropoxycalix[4]arene**

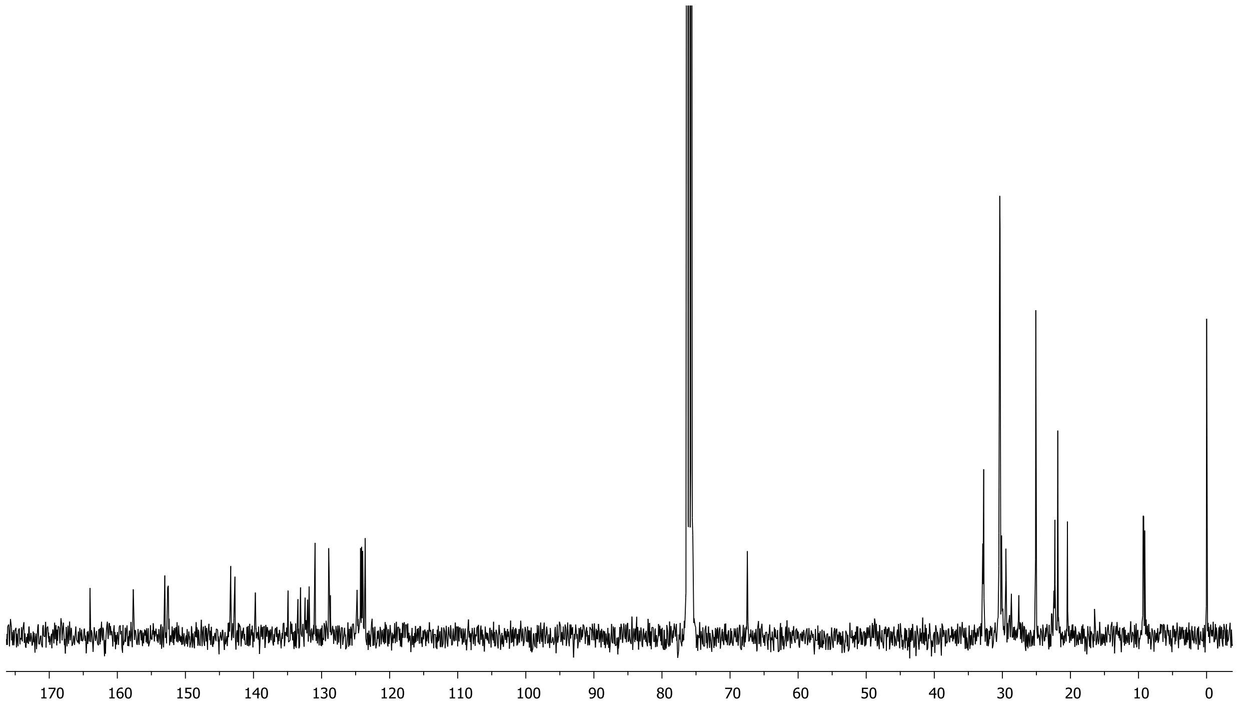


**Figure 10 – $^{13}$C NMR: (*cR*)-11,17,23-Tri-*tert*-butyl-5-((*S*)-4-*tert*-butyl-4,5-dihydrooxazol-2-yl)-4-methylthio-25,26,27,28-tetrapropoxycalix[4]arene**

**(*cS*)-11,17,23-Tri-*tert*-butyl-5-((*S*)-4-*tert*-butyl-4,5-dihydrooxazol-2-yl)-4-methylthio-25,26,27,28-tetrapropoxycalix[4]arene – (8a)**

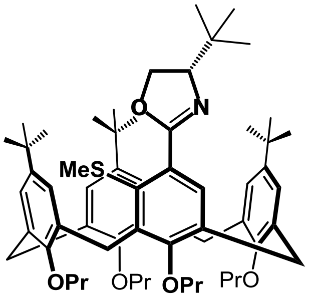


Thioether (**8a**) was synthesized according to the general ortholithiation procedure oxazoline (**4b**) (30 mg, 0.034 mmol), *t*BuLi (0.17 mmol, 5 eq), TMEDA (0.34 mmol, 10 eq), $Et_2O$ (0.2 ml) and dimethyldisulfide (0.05 ml, 5 mmol, exs) with a reaction time of 72 hours. The product was purified using silica gel column chromatography (EtOAc:PET, 1.5:98.5) yielding a white solid. IR (ATR) $cm^{-1}$: 2957 (m, –C–H stretch), 1654 (s, C=N stretch), 1480 (s, C=C stretch), 1201 (m, C–O stretch), 1008 (m, C–O stretch), 960 (m, C–H oop bend) $^1$H NMR (300 MHz, BENZENE-*d*) 0.87 – 0.99 (m, 21 H, $C(CH_3)_3$, $CH_2CH_3$), 1.23 (s, 9 H, $C(CH_3)_3$), 1.26 (s, 9 H, $C(CH_3)_3$), 1.29 (s, 9 H, $C(CH_3)_3$), 1.82 – 1.96 (m, 4 H, $CH_2CH_2CH_3$), 1.98 – 2.12 (m, 4 H, $CH_2CH_2CH_3$), 2.29 (s, 3 H, $SCH_3$), 3.12 – 3.17 (d, 1 H, *J* = 12.7 Hz, $ArCH_2Ar$), 3.27 – 3.31 (d, 2 H, *J* = 12.7 Hz, $ArCH_2Ar$), 3.66 – 3.74 (m, 1 H, $OCH_2CH_2CH_3$), 3.80 – 4.03 (m, 10 H, $OCH_2CH_2CH_3$, $OCH_2CHN$, $OCH_2CHN$), 4.51 – 4.66 (m, 5 H, $ArCH_2Ar$), 6.98 – 7.03 (m, 4 H, Ar*H*), 7.06 – 7.07 (d, 1 H, *J* = 2.4 Hz, Ar*H*) 7.37 – 7.38 (d, 1 H, *J* = 2.4 Hz, Ar*H*), 7.62 (br. s, 1 H, Ar*H*) $\delta$ ppm; $^{13}$C NMR (75 MHz, BENZENE-*d*) 10.4, 10.5, 10.6, 10.3, 22.0, 23.2, 23.5, 23.8, 23.8, 26.2, 29.7, 30.2, 31.0, 31.8, 31.8, 31.9, 32.2, 34.1, 34.2, 34.2, 34.2, 68.3, 76.8, 77.2, 77.2, 77.6, 125.2, 125.4, 125.7, 125.8, 125.9, 126.9, 128.2, 130.8, 131.3, 132.8, 132.8, 133.0, 133.9, 134.2, 134.5, 134.9, 136.0, 141.4, 144.3, 145.0, 154.3, 154.5, 154.6, 159.1, 164.4 $\delta$ ppm; $^1$H, $^1$H COSY (300/300 MHz 278 K, $C_6D_6$): $\delta^1$H/ $\delta^1$H = 2.00 / 0.88 ($CH_2CH_3$ / $CH_2CH_3$), 3.99 / 2.00 ($OCH_2CH_2$ / $CH_2CH_2$), 4.70, 4.60 / 3.36, 3.26 ($ArCH_2Ar_{eq}$ / $ArCH_2Ar_{ax}$), 7.08 / 6.96 (Ar*H* / Ar*H*) ppm; $^1$H, $^{13}$C HSQC (300/75 MHz, 278 K, $C_6D_6$) $\delta$ $^1$H/ $\delta$ $^{13}$C = 0.86 / 10.0 ($CH_2CH_3$ / $CH_2CH_3$), 0.91 / 10.0 ($CH_2CH_3$ / $CH_2CH_2$), 0.95 / 10.1 ($CH_2CH_3$ / $CH_2CH_2$), 0.96 / 25.7 ($C(CH_3)_3^{ox}$ / $C(CH_3)_3^{ox}$), 1.22 / 31.3 ($C(CH_3)_3^{Ar}$ / $C(CH_3)_3^{Ar}$), 1.25 / 31.2 ($C(CH_3)_3^{Ar}$ / $C(CH_3)_3^{Ar}$), 1.27 / 31.3 ($C(CH_3)_3^{Ar}$ / $C(CH_3)_3^{Ar}$), 1.88 / 22.8 ($OCH_2CH_2$ / $OCH_2CH_2$), 3.11 / 30.6 ($ArCH_2Ar^{eq}$ / $ArCH_2Ar^{eq}$), 3.16 / 30.4 ($ArCH_2Ar^{eq}$ / $ArCH_2Ar^{eq}$), 3.26 / 31.5 ($ArCH_2Ar^{eq}$ / $ArCH_2Ar^{eq}$), 3.30 / 31.6 ($ArCH_2Ar^{eq}$ / $ArCH_2Ar^{eq}$), 3.82 / 76.7 ($OCH_2CH_2$ /

O*C*H$_2$CH$_2$), 3.84 / 76.6 (OC*H*$_2$CH$_2$ / O*C*H$_2$CH$_2$), 3.91 / 76.6 (OC*H*$_2$CH$_2$ / O*C*H$_2$CH$_2$), 3.93 / 76.58 (OC*H*$_2$CH$_2$ / O*C*H$_2$CH$_2$), 3.99 / 67.80 (OC*H*$_2$CHN$^{ox}$ / O*C*H$_2$CHN$^{ox}$), 4.50 / 30.4 (ArC*H*$_2$Ar$^{ax}$ / Ar*C*H$_2$Ar$^{ax}$), 4.54 / 30.4 (ArC*H*$_2$Ar$^{ax}$ / Ar*C*H$_2$Ar$^{ax}$), 4.56 / 31.6 (OCH$_2$C*H*N$^{ox}$ / OCH$_2$*C*HN$^{ox}$), 4.61 / 31.6 (ArC*H*$_2$Ar$^{ax}$ / Ar*C*H$_2$Ar$^{ax}$), 4.65 / 31.5 (ArC*H*$_2$Ar$^{ax}$ / Ar*C*H$_2$Ar$^{ax}$), 6.97 / 124.9 (C$_{Ar}$*H* / *C*$_{Ar}$H), 7.05 / 125.5 (C$_{Ar}$*H* / *C*$_{Ar}$H), 7.37 / 126.43 (C$_{Ar}$*H* / *C*$_{Ar}$H), 7.60 / 130.8 (6-C$_{Ar}$*H* / 6-*C*$_{Ar}$H) ppm; $^1$H, $^{13}$C HMBC (300/75 MHz, 278 K, C$_6$D$_6$) $\delta$ $^1$H/ $\delta$ $^{13}$C 0.87 / 23.4, 77.2 (CH$_2$C*H*$_3$ / O*C*H$_2$CH$_2$, OCH$_2$*C*H$_2$), 0.89 / 23.4, 77.1 (CH$_2$C*H*$_3$ / O*C*H$_2$CH$_2$, OCH$_2$*C*H$_2$), 0.92, 23.4, 77.1 (CH$_2$C*H*$_3$ / O*C*H$_2$CH$_2$, OCH$_2$*C*H$_2$), 0.95 / 23.5, 77.1 (CH$_2$C*H*$_3$ / O*C*H$_2$CH$_2$, OCH$_2$*C*H$_2$), 0.97 / 26.1, 34.0 (C(C*H*$_3$)$_3^{ox}$ / (*C*(CH$_3$)$_3^{ox}$, OCH$_2$*C*HN$^{ox}$), 1.22 / 31.7 (C(C*H*$_3$)$_3^{Ar}$ / (*C*(CH$_3$)$_3^{Ar}$), 1.25 / 31.7, (C(C*H*$_3$)$_3^{Ar}$ / (*C*(CH$_3$)$_3^{Ar}$), 1.27 / 31.7 (C(C*H*$_3$)$_3^{Ar}$ / (*C*(CH$_3$)$_3^{Ar}$), 1.90 / 10.4, 76.8 (OCH$_2$C*H*$_2$ / CH$_2$*C*H$_3$, *C*H$_2$CH$_3$), 2.04 / 10.4, 77.1 (OCH$_2$C*H*$_2$ / CH$_2$*C*H$_3$, *C*H$_2$CH$_3$) 2.28 / 134.22 (4-SC*H*$_3$ / 4-*C*$_{Ar}$), 3.11 / 125.4, 131.2, 132.8, 136.0, 154.4, 159.1 (ArC*H*$_2$Ar$^{eq}$ / *C*$_{Ar}$H, *C*$_{Ar}$H, *C*$_{Ar}$C, *C*$_{Ar}$C, *C*$_{Ar}$O, *C*$_{Ar}$O), 3.17 / 125.4, 131.1, 135.9, 154.2, 159.2 (ArC*H*$_2$Ar$^{eq}$ / *C*$_{Ar}$H, *C*$_{Ar}$H, *C*$_{Ar}$C, *C*$_{Ar}$C, *C*$_{Ar}$O, *C*$_{Ar}$O), 3.27 / 125.6, 134.2, 154.5 (ArC*H*$_2$Ar$^{eq}$ / *C*$_{Ar}$H, *C*$_{Ar}$C, *C*$_{Ar}$O), 3.31 / 125.6, 134.3, 154.5 (ArC*H*$_2$Ar$^{eq}$ / *C*$_{Ar}$H, *C*$_{Ar}$C, *C*$_{Ar}$O), 3.84 / 10.4, 23.3, 154.2 (OC*H*$_2$CH$_2$ / CH$_2$*C*H$_3$, O*C*H$_2$CH$_3$, *C*$_{Ar}$O), 3.85 / 10.4, 23.4, 159.2 (OC*H*$_2$CH$_2$ / CH$_2$*C*H$_3$, O*C*H$_2$CH$_3$, *C*$_{Ar}$O), 3.91 / 26.0, 67.9, 164.3 (OC*H*$_2$CHN$^{ox}$ / C(*C*H$_3$)$_3$, OCH$_2$*C*HN$^{ox}$, O*C*N), 4.50 / 125.3, 131.23, 134.0, 154.3, 159.1 (ArC*H*$_2$Ar$^{ax}$ / *C*$_{Ar}$H, *C*$_{Ar}$H, *C*$_{Ar}$C, *C*$_{Ar}$C, *C*$_{Ar}$O, *C*$_{Ar}$O) 4.54 / 125.3, 132.7, 135.9, 142.5, 154.3, 159.2 (ArC*H*$_2$Ar$^{ax}$ / *C*$_{Ar}$H, *C*$_{Ar}$H, *C*$_{Ar}$C, *C*$_{Ar}$C, *C*$_{Ar}$O, *C*$_{Ar}$O), 6.97 / 32.0, 123.1, 154.5 (Ar*H* / C(*C*H$_3$)$_3$, *C*$_{Ar}$H, *C*$_{Ar}$O), 6.99 / 34.1, 125.7, 154.4 (Ar*H* / C(*C*H$_3$)$_3$, *C*$_{Ar}$H, *C*$_{Ar}$O), 7.03 / 32.1, 125.4, 154.3 (Ar*H* / C(*C*H$_3$)$_3$, *C*$_{Ar}$H, *C*$_{Ar}$O), 7.05 / 31.7, 125.2, 154.5 (Ar*H* / C(*C*H$_3$)$_3$, *C*$_{Ar}$H, *C*$_{Ar}$O), 7.35 / 125.6, 154.4(Ar*H* / *C*$_{Ar}$H, *C*$_{Ar}$O), 7.60 / 30.9, 134.2, 159.12, 164.37 (6-Ar*H* / Ar*C*H$_2$Ar, 4-*C*SCH$_3$, *C*$_{Ar}$O, O*C*N) ppm; HRMS-TOF MS ESI+: *m/z* [M+H]$^+$ calculated for $C_{60}H_{86}NO_5S$ 932.6227; found: 932.6257

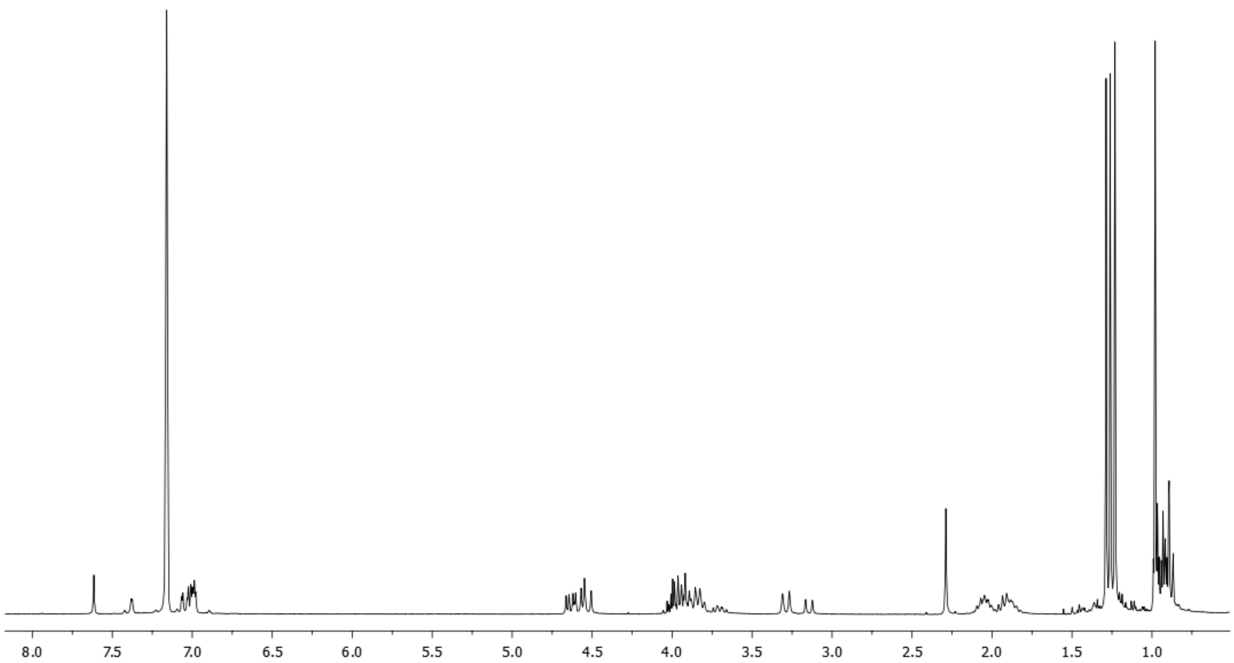


**Figure 11 – $^{1}$H NMR: (*cS*)-11,17,23-Tri-*tert*-butyl-5-((*S*)-4-*tert*-butyl-4,5-dihydrooxazol-2-yl)-4-methylthio-25,26,27,28-tetrapropoxycalix[4]arene**

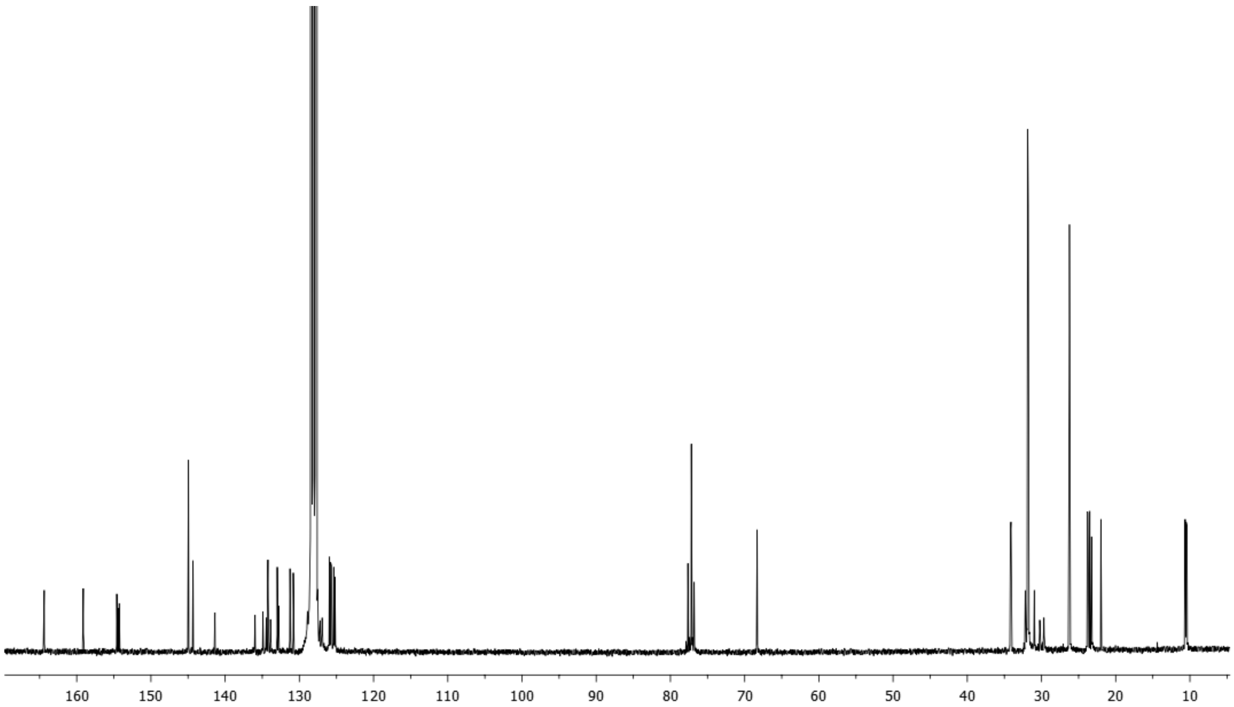


**Figure 12 – $^{13}$C NMR: (*cS*)-11,17,23-Tri-*tert*-butyl-5-((*S*)-4-*tert*-butyl-4,5-dihydrooxazol-2-yl)-4-methylthio-25,26,27,28-tetrapropoxycalix[4]arene**

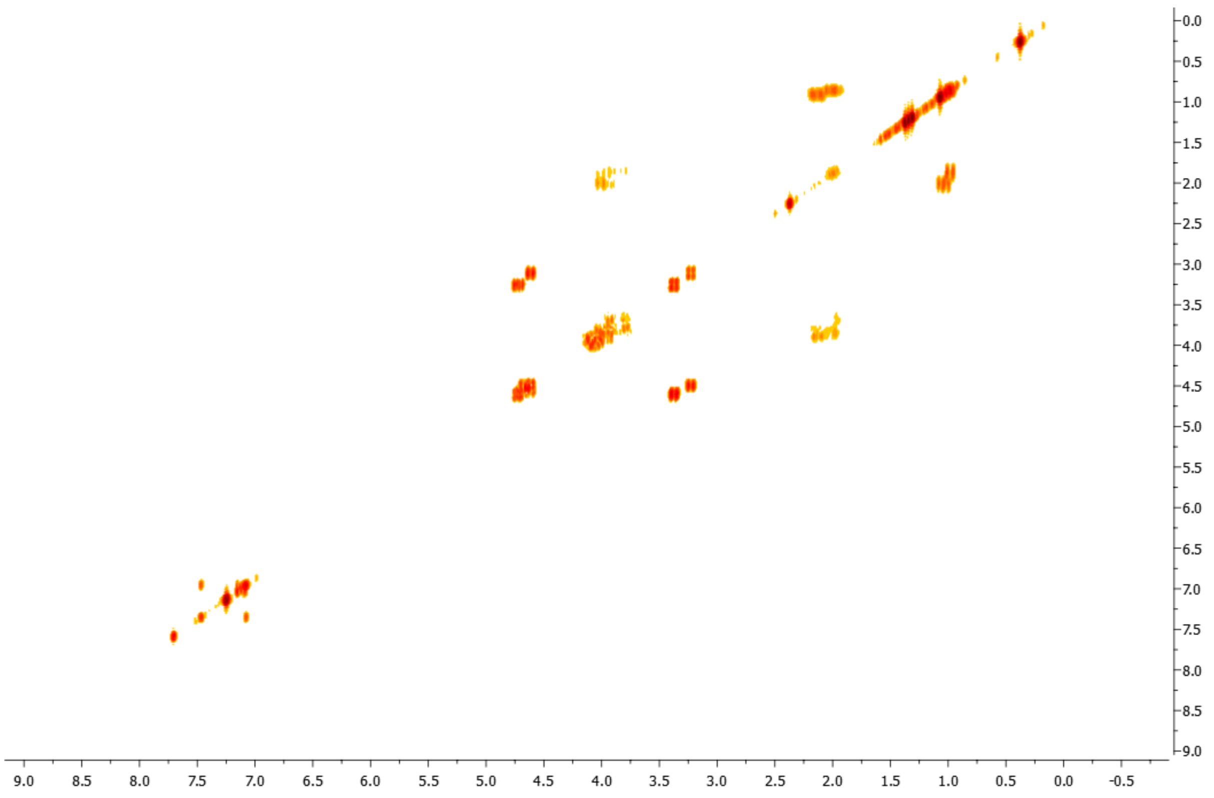


**Figure 13 – COSY 2D NMR: (*cS*)-11,17,23-Tri-*tert*-butyl-5-((*S*)-4-*tert*-butyl-4,5-dihydrooxazol-2-yl)-4-methylthio-25,26,27,28-tetrapropoxycalix[4]arene**

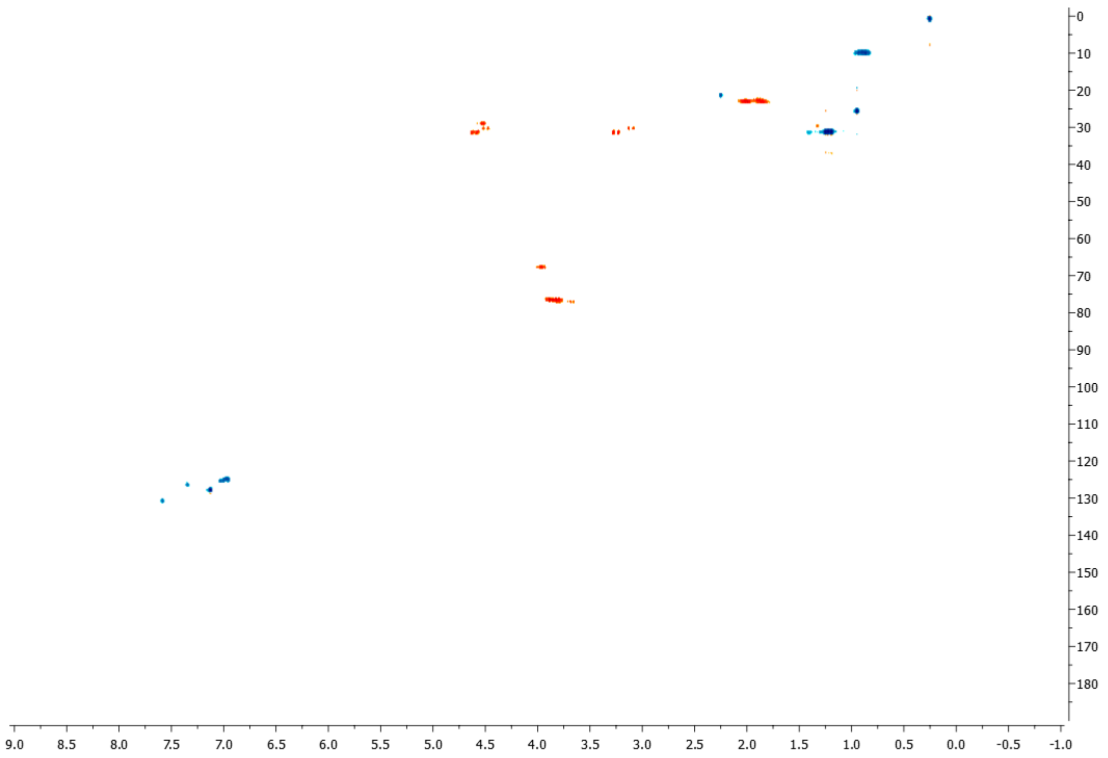


**Figure 14 – HSQC 2D NMR: (*cS*)-11,17,23-Tri-*tert*-butyl-5-((*S*)-4-*tert*-butyl-4,5-dihydrooxazol-2-yl)-4-methylthio-25,26,27,28-tetrapropoxycalix[4]arene**

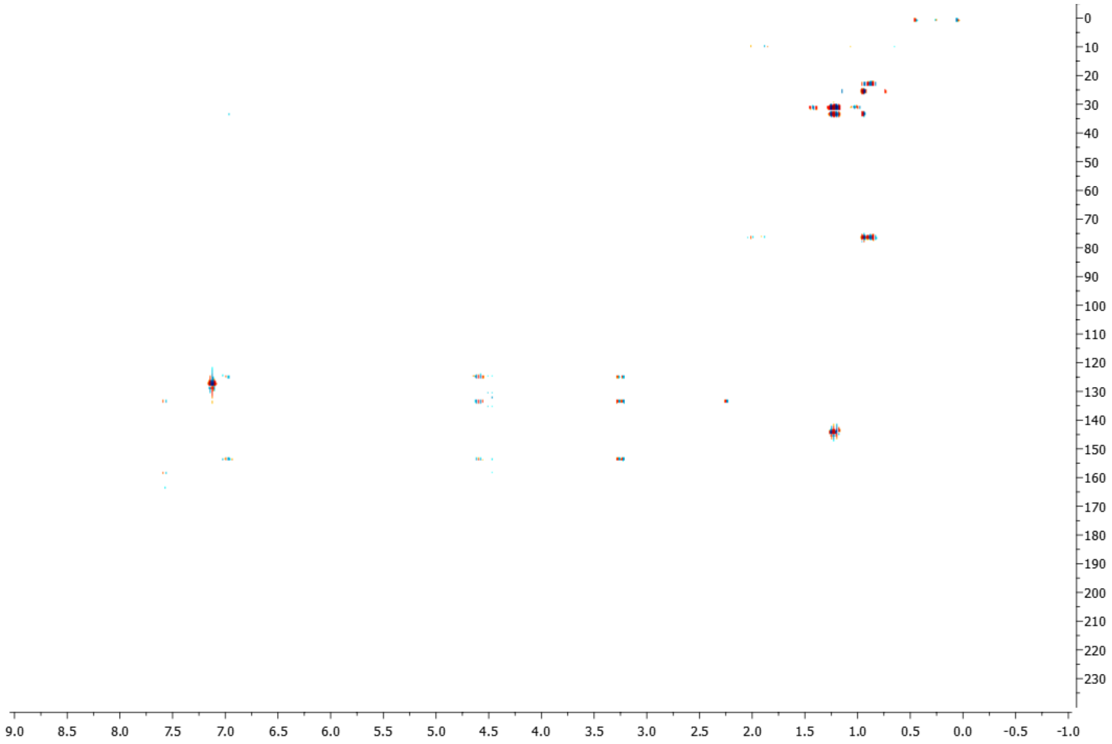

**Figure 15 – HMBC 2D NMR: (*cS*)-11,17,23-Tri-*tert*-butyl-5-((*S*)-4-*tert*-butyl-4,5-dihydrooxazol-2-yl)-4-methylthio-25,26,27,28-tetrapropoxycalix[4]arene**

**(cS)-5-((S)-4-Isopropyl-4,5-dihydrooxazol-2-yl)-4-methylthio-25,26,27,28-tetrapropoxycalix[4]arene – (5a)**

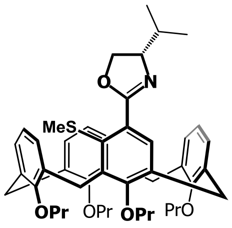


Thioether (**5a**) was synthesized according to the general ortholithation procedure from oxazoline (**1a**) (20 mg, 0.24 mmol), *s*BuLi (1.20 mmol, 5 eq), di-*t*Bu-diglyme (0.06 ml, 0.14 mmol, 10 eq), pentane (5.0 ml) and dimethyldisulfide (0.05 ml, exs) with a reaction time of 7 hours. Excess ligand was removed under high vacuum at 65 °C over a period of 48 h. Conversion by $^{1}$H NMR 96 %. Selectivity by 1H NMR spectroscopy = 9:1; $R_f$ = 0.54 (EtOAc:PET, 10:90); IR (film) cm$^{-1}$: 2958 (w, -C-H stretch), 2920 (w, -C-H stretch) 2872 (Methylene stretch), 1651 (w, C=N Stretch), 1452 (s, C=C stretch), 1191 (s, C-O stretch), 1006 (s, C-O stretch), 963 (s, C-H oop bend), 755 (s, C-H oop bend); $^{1}$H NMR (300 MHz, CHLOROFORM-*d*) 0.85 - 0.92 (m, 6 H, $CH_2CH_3$), 1.03 (d, *J* = 6.7 Hz, 3 H, $CH(CH_3)_2$), 1.06 - 1.14 (m, 9 H, $CH_2CH_3$, $CH(CH_3)_2$), 1.81 - 2.02 (m, 9 H, $CH_2CH_2CH_3$, $CH(CH_3)_2$), 2.41 (s, 3 H, $SCH_3$), 3.10 - 3.19 (m, 3 H, $ArCH_2Ar$), 3.61 - 3.75 (m, 4 H, $OCH_2CH_2$), 3.95 - 4.06 (m, 4 H, $OCH_2CH_2$), 4.14 - 4.24 (m, 2 H, $ArCH_2Ar$, $OCH_2CHN$), 4.29 - 4.33 (m, 2 H, $OCH_2CHN$), 4.36 - 4.50 (m, 4 H, $ArCH_2Ar$), 5.98 (d, *J* = 7.3 Hz, 1 H, Ar*H*), 6.04 (d, *J* = 7.3 Hz, 1 H, Ar*H*), 6.08 - 6.18 (m, 3 H, Ar*H*), 6.22 (d, *J* = 7.3 Hz, 1 H, Ar*H*), 6.91 (t, *J* = 7.3 Hz, 1 H, Ar*H*), 7.10 (d, *J* = 7.4 Hz, 2 H, Ar*H*), 7.32 (s, 1 H, Ar*H*) δ ppm; $^{13}$C NMR (75 MHz, CHLOROFORM-d) 10.0, 11.0, 18.6, 19.4, 21.5, 23.1, 23.7, 27.6, 29.9, 30.9, 31.1, 31.2, 33.0, 70.4, 73.3, 76.7, 76.9, 77.1, 77.4, 121.9, 122.3, 122.4, 127.2, 127.4, 127.7, 127.8, 129.0, 129.1, 130.0, 132.2, 133.0, 133.2, 133.5, 133.7, 137.2, 137.3, 137.7, 142.5, 155.2, 155.3, 158.2, 160.3, 165.3 δ ppm; HRMS– TOF MS ES$^{+}$: m/z $[M+H]^{+}$ calcd for $C_{47}H_{59}NO_5S$: 750.4192; found: 750.4227.

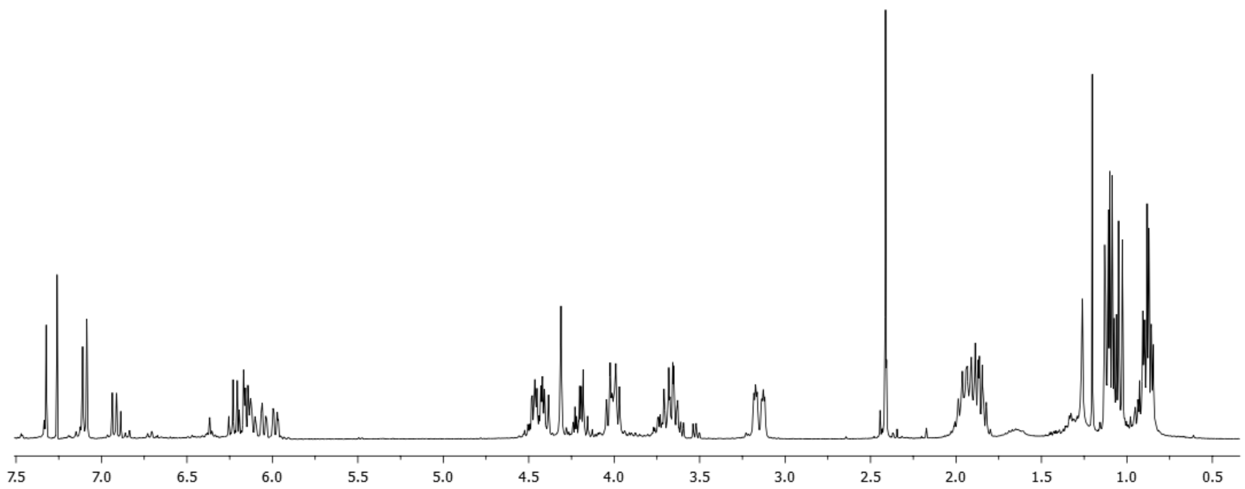


**Figure 16 – $^{1}$H NMR: (*cS*)-5-((*S*)-4-Isopropyl-4,5-dihydrooxazol-2-yl)-4-methylthio- 25,26,27,28-tetrapropoxycalix[4]arene**

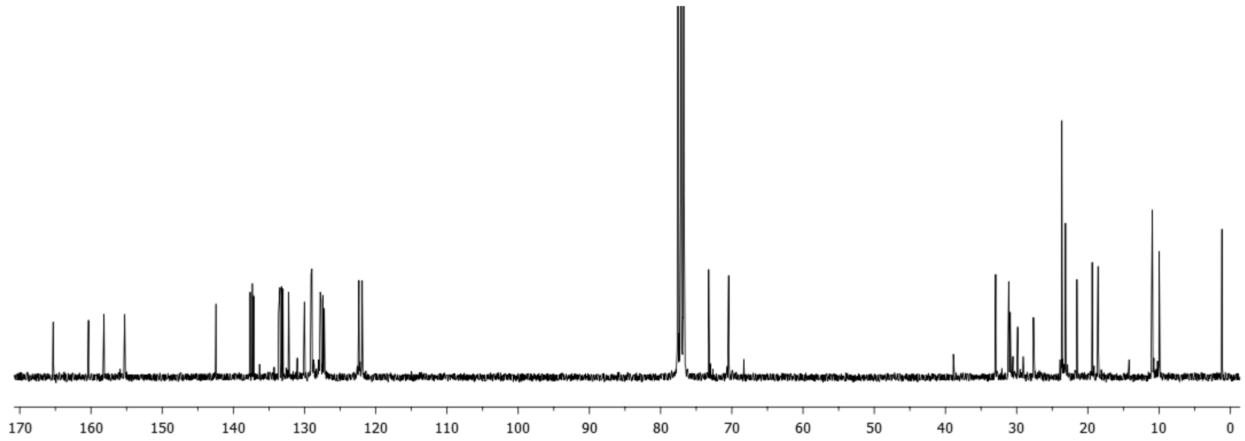


**Figure 17 – $^{13}$C NMR: (*cS*)-5-((*S*)-4-Isopropyl-4,5-dihydrooxazol-2-yl)-4-methylthio-25,26,27,28-tetrapropoxycalix[4]arene**

## 3. Synthesis of 'flat' model ligand compounds.

**2-(4-Methoxyphenyl)-4,5-dihydrooxazole – (10a)**

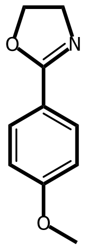

4-methoxybenzoic acid (100 mg, 0.71 mmol) was added to dry DCM (50 ml) in an oven dried flask under argon. Oxalyl chloride (0.30 ml, 3.55 mmol) was added and the mixture was stirred overnight. The excess $(COCl)_2$ and DCM were removed under reduced pressure. Ethanolamine (0.10 ml, 101.6 mg, 1.66 mmol) and dry DCM (20 ml) were added to an oven dried flask, followed by $Et_3N$ (1.5 ml) and the mixture was cooled (0 °C). The acid chloride was added to dry DCM (20 ml) and added drop wise to the amino alcohol mixture using a syringe pump (35 ml/h). Once all the acid chloride had been added, the mixture was allowed to warm to room temperature and stirred for two hours. The excess solvent was then removed under reduced pressure, yielding the crude amide product. The amide was then again added to dry DCM (20 ml). Mesyl chloride (0.16 ml, 2.13 mmol) and $Et_3N$ (0.4 ml, 2.84 mmol) were then added and the progress of the reaction was monitored by TLC. Once complete the solvent was removed under reduced pressure and the crude product was purified by silica gel chromatography (EtOAc:PET, 10:90), yielding an off-white solid (109 mg, 0.61 mmol, 87%). Mp 60 - 62 °C (Lit.[3] 63 °C); $R_f$ = 0.22 (EtOAc:PET, 40:60); $^1$H NMR (300 MHz, $CDCl_3$, matches literature[4]) 3.85 (s, 3 H, OC*H*$_3$), 4.01 (t, *J* = 9.9 Hz, 2 H, NC*H*$_2$CH$_2$O), 4.38 (t, *J* = 9.9 Hz, 2 H, NCH$_2$C*H*$_2$O), 6.90 (d, *J* = 8.9 Hz, 2 H, Ar*H*), 7.88 (d, *J* = 8.9 Hz, 2 H, Ar*H*) δ ppm; $^{13}$C NMR (75 MHz, $CDCl_3$) 54.8 (N*C*H$_2$CH$_2$O), 55.3 (O*C*H$_3$), 67.5 (NCH$_2$*C*H$_2$O), 113.7 (*C*Ar), 120.3 (*C*Ar), 129.8 (*C*Ar), 162.0 (*C*Ar), 164.4 (O*C*(Ar)=N) δ ppm.

**(*S*)-4-Isopropyl-2-(4-methoxyphenyl)-4,5-dihydrooxazole – (11a)**

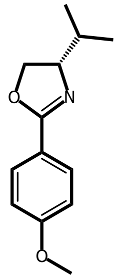

(**11a**) was prepared in a procedure analogous to that used for (**10a**). 4-methoxybenzoic acid (100 mg, 0.71 mmol) and L-valinol (89 mg, 0.86 mmol). Purification was achieved by silica gel chromatography (EtOAc:PET, 10:90), yielding a beige solid (112 mg, 0.51 mmol, 72%). Mp 54 - 57 °C; $R_f$ = 0.56 (EtOAc:PET, 40:60); $[\alpha]_D^{24}$ = – 66.5° (*c* 1.03, DCM); $^1$H NMR (300 MHz, $CDCl_3$, corresponds to literature[5]) 0.98 (d, *J* = 6.8 Hz, 3 H, CH(C*H*$_3$)$_2$), 1.08 (d, *J* = 6.8 Hz, 3 H, CH(C*H*$_3$)$_2$), 1.86 (d, *J* = 6.8 Hz, 1 H, C*H*(CH$_3$)$_2$), 3.84 (s, 3 H, OC*H*$_3$), 4.06 - 4.09 (m, 1 H, NC*H*), 4.34 - 4.36 (m, 1 H, OC*H*$_2$CH), 4.37 - 4.39 (m, 1 H, OC*H*$_2$CH), 6.74 (dd, *J* = 8.6, 2.5 Hz, 2H, Ar*H*), 7.87 (dd, *J* = 8.6, 2.5 Hz, 2 H, Ar*H*) δ ppm; $^{13}$C NMR (75 MHz, $CDCl_3$) δ ppm 19.5 (CH(*C*H$_3$)$_2$), 32.6 (*C*H(CH$_3$)$_2$), 55.9 (O*C*H$_3$), 71.9 (O*C*H$_2$), 75.7 (N*C*H), 111.2 (*C*Ar), 122.0 (*C*Ar), 130.4 (*C*Ar), 161.5 (*C*Ar), 165.8 (O*C*(Ar)=N).

**(*S*)-4-*tert*-Butyl-2-(4-methoxyphenyl)-4,5-dihydrooxazole – (12a)**

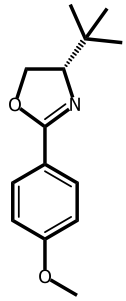

(**12a**) was prepared in a procedure analogous to that used for (**10a**). 4-methoxybenzoic acid (500 mg, 3.56 mmol) and L-*tert*-leucinol (428 mg, 3.66 mmol). Purification was achieved by silica gel column chromatography (EtOAc:PET, 10:90), yielding an off-white solid (598 mg, 2.56 mmol, 72%). Mp 53 - 55 °C; $R_f$ = 0.66 (EtOAc:PET, 40:60); $[\alpha]_D^{24}$ = –18.8° (*c* 0.25, DCM); $^1$H NMR (300 MHz, $CDCl_3$) 0.89 (s, 9 H, C(C*H*$_3$)$_3$), 3.85 (s,

3 H, OC$H_3$), 4.02 (dd, $J$ = 10.0, 7.5 Hz, 1 H, NC$H$), 4.21 (dd, $J$ = 8.7, 7.5 Hz, 1 H, OC$H$), 4.32 (dd, $J$ = 10.0, 8.5 Hz, 1 H, OC$H$), 6.89 (d, $J$ = 8.9 Hz, 2 H, Ar$H$), 7.89 (d, $J$ = 8.9 Hz, 2 H, Ar$H$) δ ppm; $^{13}$C NMR (75 MHz, $CDCl_3$) 25.9 (C($C$H$_3$), 34.0 ($C$(CH$_3$), 55.3 (O$C$H$_3$), 68.6 (O$C$H$_2$), 76.1 (N$C$H), 113.6 ($C$Ar), 120.5 ($C$Ar), 129.9 ($C$Ar), 161.9 ($C$Ar), 163.0 (O$C$(Ar)=N) δ ppm.

**2-(4-Methoxy-2-(methylthio)phenyl)-4,5-dihydroxazole – (10)**

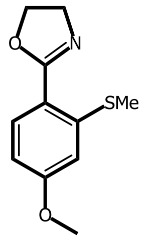

Oxazoline (**10a**) (41 mg, 0.176 mmol) was added to dry THF (3.5 ml) in oven dried Schlenk under argon. *i*PrLi (0.48 ml, 0.264 mmol, 1.5 eq) was carefully added to the stirring mixture. The mixture was stirred for 1.5 hours at −78 °C. Dimethyl disulfide (0.10 ml, 1.13 mmol, exs) was added and the mixture was stirred at −78 °C for a further 30 minutes. The solution was then allowed to warm to room temperature. After 2 hours, the mixture was finally quenched by the addition of $H_2O$ (2.0 ml). The crude product was extracted using EtOAc (2 × 40 ml). The combined organic layers were dried over $MgSO_4$ and the solvent was removed under reduced pressure. To remove any remaining dimethyl disulfide, the residue was dissolved in methanol and the solvent once again removed. Yield by $^1$H NMR 73%. Further purification was achieved by silica gel column chromatography (EtOAc:PhMe, 20:80) to yield (**10a**) as a yellow oil (28 mg, 0.125 mmol, 71%). $R_f$ = 0.58 (EtOAc:PhMe, 40:60); $^1$H NMR (300 MHz, $CDCl_3$) 2.46 (s, 3 H, SC$H_3$), 3.87 (s, 3 H, OC$H_3$), 4.13-4.20 (m, 2 H, NC$H_2$CH$_2$O), 4.32-4.39 (m, 2 H, NCH$_2$C$H_2$O), 6.66 (dd, $J$ = 8.6, 2.4 Hz, 1 H, Ar$H$), 6.77 (d, $J$ = 2.4 Hz, 1 H, Ar$H$), 7.83 (d, $J$ = 8.6 Hz, 1 H, Ar$H$) δ ppm; $^{13}$C NMR (75 MHz, $CDCl_3$) 14.7 (S$C$H$_3$), 55.6 (O$C$H$_3$), 67.5 (O$C$H$_2$), 68.2 (N$C$H), 106.6 ($C$Ar), 110.4 ($C$Ar), 127.2 ($C$Ar), 128.6 ($C$Ar), 130.4 ($C$Ar), 142.9 ($C$Ar), 165.1 (O$C$(Ar)=N) δ ppm.

**(*S*)-4-Isopropyl-2-(4-methoxy-2(methylthio)phenyl)-4,5-dihydrooxazole – (11)**

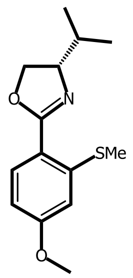

(**11**) was prepared in a procedure analogous to that used for (**10**) from oxazoline (**11a**) (41 mg, 0.187 mmol). Yield by $^{1}$H NMR 83%. Further purification was achieved by silica gel column chromatography (EtOAc:PhMe, 20:80) to yield (**11**) as a yellow-orange oil (40 mg, 0.150 mmol, 80%). $R_f$ = 0.47 (EtOAc:PhMe, 30:70); $[\alpha]_D^{24}$ = –70.2° (*c* 0.16, DCM); $^{1}$H NMR (400 MHz, $CDCl_3$) 0.95 (d, *J* = 6.8 Hz, 3H, CH(C$H_3$)$_2$), 1.06 (d, *J* = 6.8 Hz, 3H, CH(C$H_3$)$_2$), 1.84 (d, *J* = 6.8 Hz, 1H, C*H*($CH_3$)$_2$), 2.44 (s, 3H, SC$H_3$), 3.86 (s, 3H, OC$H_3$), 4.06 - 4.09 (m, 1H, NC*H*), 4.15 - 4.21 (m, 1H, OC$H_2$CH), 4.31 - 4.35 (m, 1H, OC$H_2$CH), 6.65 (dd, *J* = 8.6, 2.5 Hz, 1H, Ar*H*), 6.76 (d, *J* = 2.5 Hz, 1H, Ar*H*), 7.77 (d, *J* = 8.6 Hz, 1H, Ar*H*) δ ppm; $^{13}$C NMR (100 MHz, $CDCl_3$) 15.9 (S$CH_3$), 19.7 (CH($CH_3$)$_2$), 33.5 (*C*H($CH_3$)$_2$), 55.7 (O$CH_3$), 67.5 (O$CH_2$), 69.2 (N*C*H), 107.6 (*C*Ar), 110.6 (*C*Ar), 124.3 (*C*Ar), 130.0 (*C*Ar), 132.6 (*C*Ar), 158.9 (*C*Ar), 162.1 (O*C*(Ar)=N) δ ppm.

**(*S*)-4-*tert*-Butyl-2-(4-methoxy-2-(methylthio)phenyl)-4,5-dihydrooxazole – (12)**

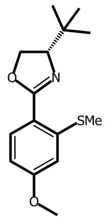

(**12**) was prepared in a procedure analogous to that used for (**10**) from oxazoline (**12a**) (41 mg, 0.176 mmol). Yield by $^{1}$H NMR 89%. Further purification was achieved by silica gel column chromatography (EtOAc:PhMe, 20:80) to yield (**12**) as a yellow oil (43 mg, 0.153 mmol, 87%). $R_f$ = 0.39 (EtOAc:PhMe, 20:80); $[\alpha]_D^{24}$ = –122.4° (*c* 0.40, DCM); $^{1}$H NMR (400 MHz, $CDCl_3$) 0.99 (s, 9 H, C(C$H_3$)$_3$), 2.43 (s, 3 H, SC$H_3$), 3.86 (s, 3 H, OC$H_3$), 3.93 - 4.34 (m, 3 H, OC$H_2$C*H*N), 6.64 (dd, *J* = 8.6, 2.5 Hz, 1 H, Ar*H*), 6.76 (d, *J* = 2.5 Hz, 1 H, Ar*H*), 7.75 (d, *J* =

8.6 Hz, 1 H, Ar*H*) δ ppm; $^{13}$C NMR (100 MHz, $CDCl_3$) 15.6 ($SCH_3$), 25.5 (C($CH_3$)$_3$), 33.8 (*C*($CH_3$)$_3$), 55.0 (O*C*$H_3$), 67.4 (O*C*$H_2$), 68.3 (N*C*H), 107.5 (*C*Ar), 110.4 (*C*Ar), 113.2 (*C*Ar), 129.6 (*C*Ar), 131.4 (*C*Ar), 143.0 (*C*Ar), 161.1 (O*C*(Ar)=N) δ ppm.

## 4. Asymmetric catalytic study: Materials and results.

1,3-diphenylprop-2-enyl acetate (**13**),[6] *N,O*-bis(trimethylsilyl)acetamide (BSA)[7] and the allyl palladium(II) chloride dimer[8] were prepared according to literature procedures.

***General procedure for palladium-catalysed allylic substitutions***[9]

[Pd($\eta^3$-$C_3H_5$)Cl]$_2$ (4 mg, 0.01 mmol, 2.5 mol %) was added to an oven-dried Schlenk flask under argon. A solution of the appropriate ligand (0.04 mmol, 10 mol%) in dry DCM (1.0 ml) was added and the resulting mixture was stirred for 30 minutes at room temperature. (**13**) (100 mg, 0.4 mmol) in dry DCM (1.0 ml) was then added, followed by dimethyl malonate (0.14 ml, 1.4 mmol 3.5 eq), BSA (0.30 ml, 1.2 mmol, 3 eq) and lithium acetate (2 mg, 0.02 mmol, 0.5 eq). The reaction mixture was stirred at room temperature until complete consumption of starting material was observed by TLC. The solution was diluted with DCM (20 ml) and poured into ice-cold saturated aqueous $NH_4Cl$ (50 ml). The layers were separated and the aqueous phase extracted with DCM (3 × 30 ml). The combined organic layers were dried over $MgSO_4$ before the solvent was removed under reduced pressure. The residue was purified by silica gel column chromatography (EtOAc:PET, 10:90) to yield the product (*E*)-dimethyl 2-(1,3-diphenylallyl)malonate (**14**) as a colourless oil. $R_f$ = 0.30 (EtOAc:PET, 10:90); $^1$H NMR (300 MHz, $CDCl_3$, corresponds to literature[6]) 3.40 (s, 3 H, $CO_2CH_3$), 3.76 (s, 3 H, $CO_2CH_3$), 3.94 (d, *J* = 10.8 Hz, 1 H, CH($CO_2CH_3$)$_2$), 4.24 (dd, *J* = 10.8, 8.5 Hz, 1 H, PhC*H*CH($CO_2CH_3$)$_2$), 6.29 (dd, *J* = 15.9, 8.5 Hz, 1 H, *H*C=CHPh), 6.46 (d, *J* = 15.9 Hz, 1 H, HC=C*H*Ph), 7.07-7.44 (m, 10 H, Ar*H*) δ ppm; $^{13}$C NMR (75 MHz, $CDCl_3$) 31.1 (Ph*C*HCH($CO_2$Me)$_2$), 52.5 (O*C*$H_3$), 57.6 (*C*H($CO_2$Me)$_2$), 125.8-131.8 (*C*Ar, *C*=*C*), 166.9 (*C*$O_2$Me) δ ppm.

*HPLC conditions and results.*

Chiralpak IC (250 × 4.6 mm, 5 μm), 3 ml/min, 40 °C, UV (254 nm), Hexane:isopropanol 95:5. $Rt_1$: 5.83 (R), $Rt_2$: 6.56 (S).

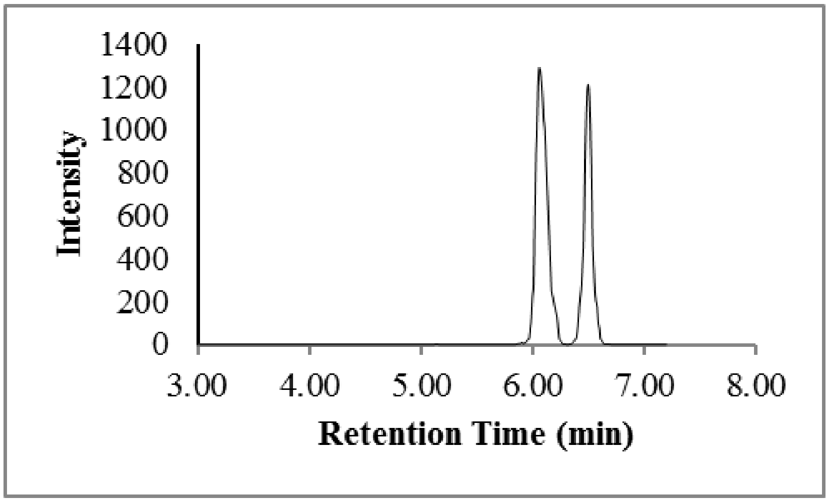


**Figure 18: HPLC chromatogram: ligand (10)**

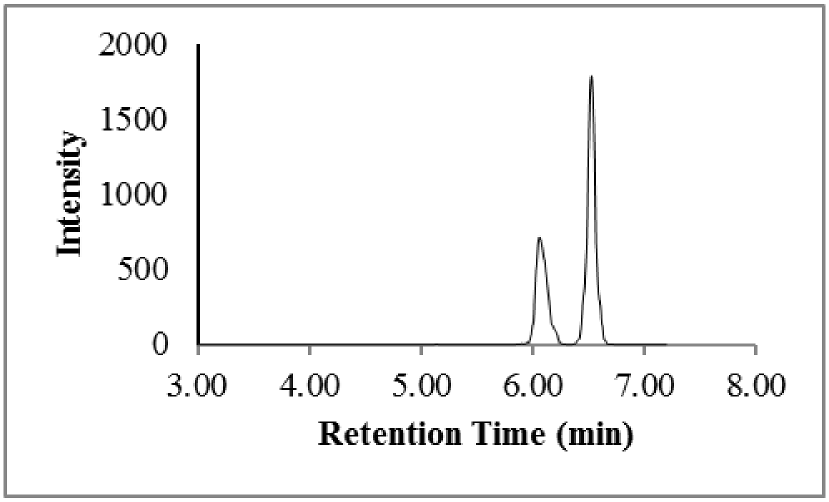


**Figure 19: HPLC chromatogram: ligand (11)**

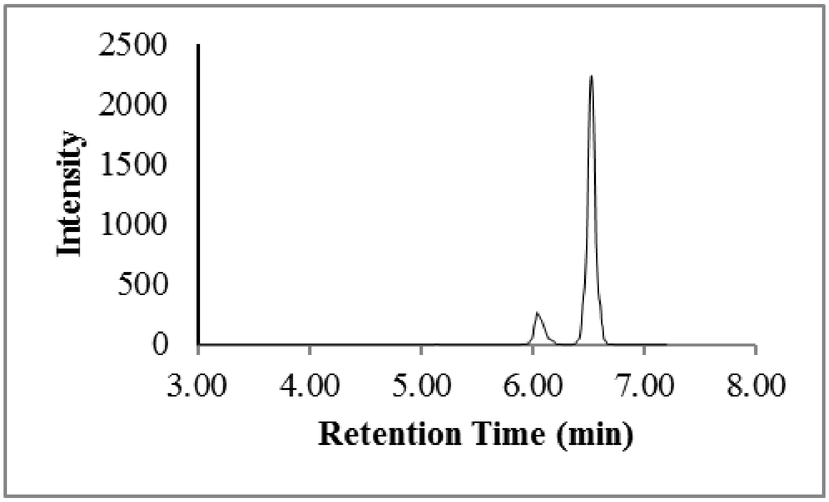


**Figure 20: HPLC chromatogram: ligand 12**

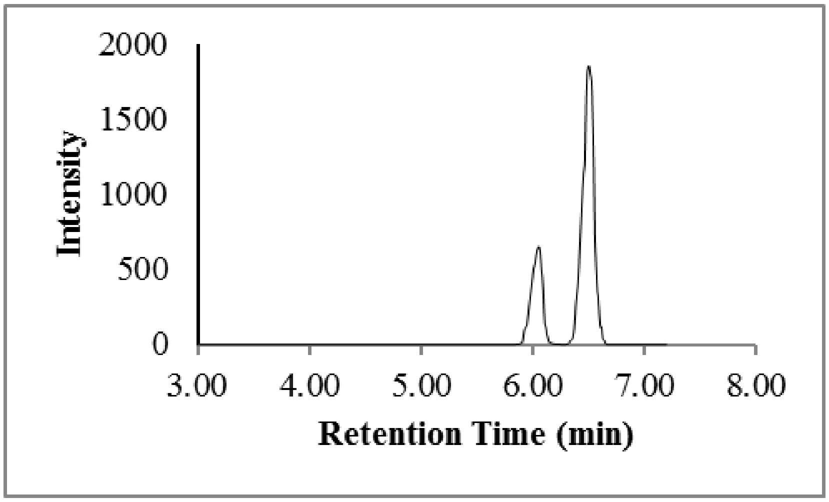


**Figure 21: HPLC chromatogram: ligand (5a)**

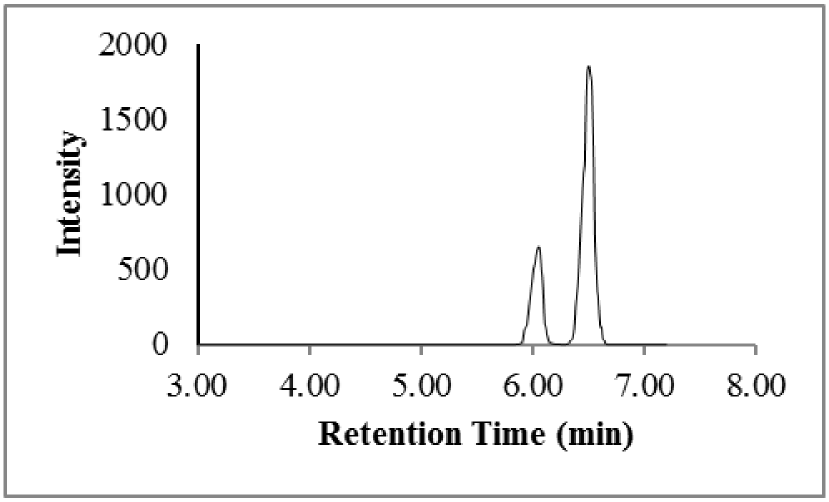


**Figure 22: HPLC chromatogram: ligand (5b)**

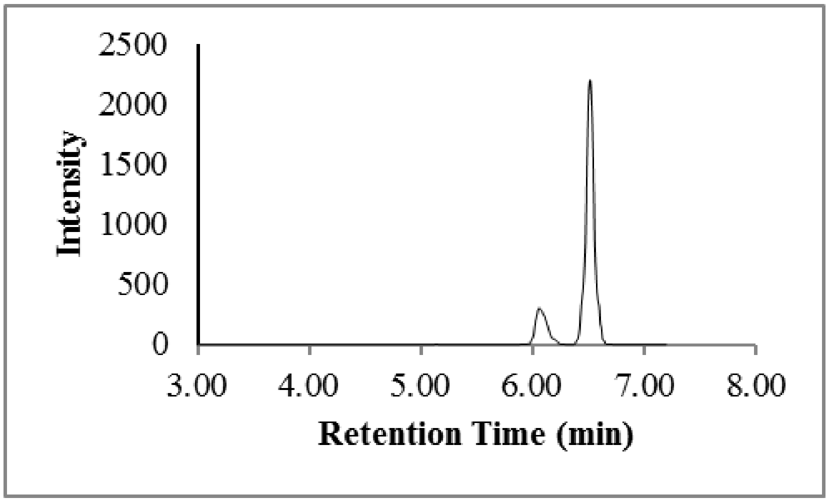


**Figure 23: HPLC chromatogram: ligand (6a)**

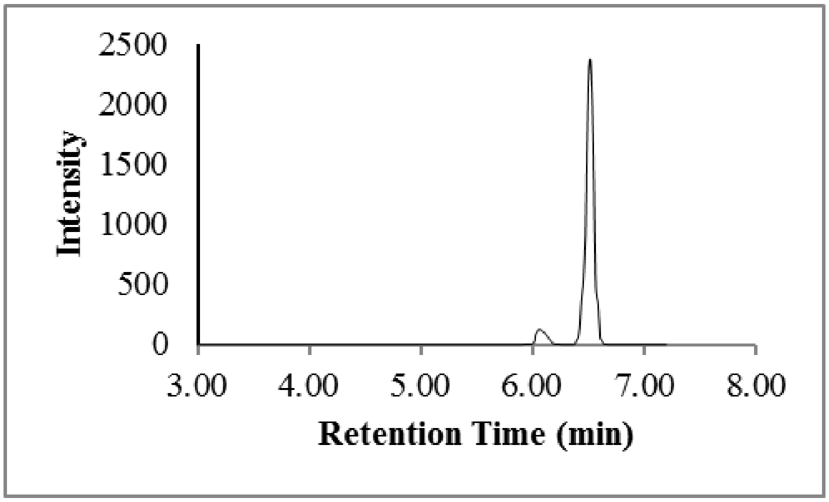


**Figure 24: HPLC chromatogram: ligand (6b)**

## 5. Identification of the major/minor diastereomers for the *t*-BuOx calixarene

Identification of the major diastereomer lies in past work on the isopropyl oxazoline calixarenes.[1] Scheme 1 shows the standard ortholithiation conditions for these compounds. It had been shown that the major diastereomer produced with these reaction conditions was in the *cR* configuration by X-ray diffraction. However, after numerous unsuccessful recrystallization attempts of *tert*-butyl oxazoline lithiation products a new approach was taken. Scheme 1 shows the standard ortholithiation conditions of calixarene **1a**. Quenching with dibromoethane followed by hydrolysis of the oxazoline yielded the bromo acid product in the *cR* configuration. The same reaction conditions, but this time with oxazoline **4a**, again yielded the bromo acid. The configuration of this product (*cR*) was confirmed by comparing the $[\alpha]_D$ values of the two separate reactions. The $[\alpha]_D$ for the bromo acid from scheme 1 had a value of –11.2°, which corresponded well with the $[\alpha]_D$ value of −10.8° recorded for the bromo acid obtained in scheme 2, confirming the identical configuration for both compounds.

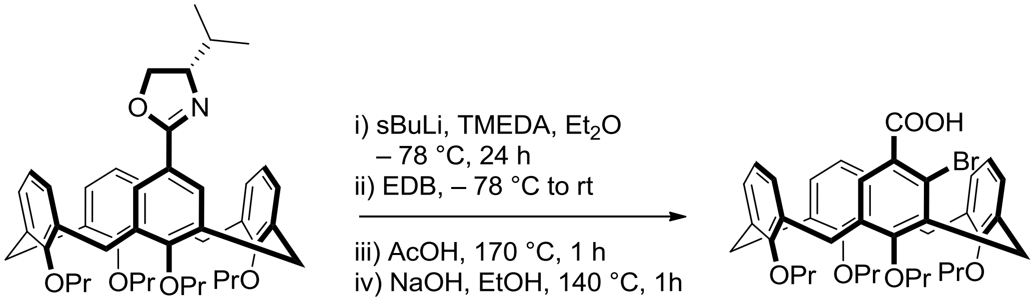


**Scheme 1: The ortholithiation and subsequent hydrolysis of calixarene (1a), yielding (cR)-4-bromo-carboxy-25,26,27,28-tetrapropoxycalix[4]arene**

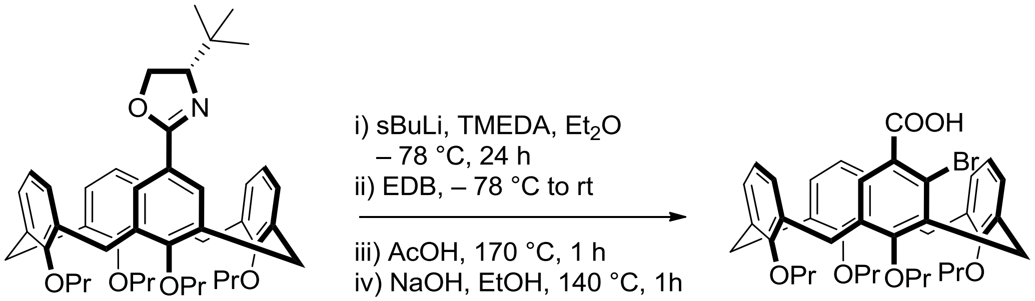


**Scheme 2: The ortholithiation and subsequent hydrolysis of caxliarene (4a), yielding (cR)-4-bromo-carboxy-25,26,27,28-tetrapropoxycalix[4]arene**

**($c$R)-4-Bromo-5-carboxy-25,26,27,28-tetrapropoxycalix[4]arene**

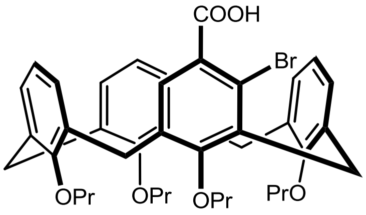


Bromo oxazoline calixarene (19.0 mg, 0.024 mmol) was added to a 2.5 ml microwave reaction vessel. Glacial acetic acid (1.5 ml) and water (0.6 ml) were then added. The flask was heated to 170 °C for 1 h, after which the excess solvent was removed under reduced pressure affording an oil as the crude product. Water (1.0 ml), ethanol (1.0 ml) and lithium hydroxide (200 mg, 8.3 mmol, exs) were then added to flask and it was heated to 140 °C for 1 h. The contents of the flask was then transferred to a separating funnel. The product was extracted using DCM (2 × 30 ml). The organic layer was washed with 1M HCl (20 l) and dried over $MgSO_4$, after which the excess solvent was removed under reduced pressure. The product was achieved using silica gel chromatography, yielding a white foam (13 mg, 0.018 mmol, 75 % yield). Mp 151 – 155 °C (foam); $R_f$ = 0.67 (EtOAc:PET, 32:68); $[\alpha]_D^{24.5}$ = -11.2 (c, 1.0 $CHCl_3$); IR (film) $cm^{-1}$: 2960 (s, -C-H stretch), 1696 (s, C=N stretch), 1454 (s, C=C stretch), 1206 (m, C-O stretch), 966 (m, C-H, oop bend), 758 (s, C-H, oop bend): $^1$H NMR (300 MHz, CHLOROFORM-*d*) 0.83 (m, 6 H, $CH_2CH_3$), 1.02 (t, $J$ = 7.3 Hz, 6 H, $CH_2CH_3$), 1.72 – 1.98 (m, 8 H, $CH_2CH_2CH_3$), 3.08 (d, $J$ = 13.4 Hz, 2 H, Ar$CH_2$Ar), 3.15 (d, $J$ = 13.9 Hz, 1 H, Ar$CH_2$Ar), 3.54 – 3.66 (m, 4 H, $OCH_2CH_2$), 3.87 – 4.16 (m, 5 H, $OCH_2CH_2$, Ar$CH_2$Ar), 4.33 (d, $J$ = 13.9 Hz, 1 H, Ar*H*), 4.33 (d, $J$ = 13.4 Hz, 3 H, Ar$CH_2$Ar), 6.00 – 6.23 (m, 6 H,Ar*H*), 7.02 (d, $J$ = 7.5 Hz, 2 H, Ar*H*), 7.70 (s, 1 H, Ar*H*) $\delta$ ppm; $^{13}$C NMR (75 MHz, CHLOROFORM-*d*) 9.8, 9.8, 10.7, 10.8, 23.0, 23.0, 23.1, 23.4, 23.5, 29.6, 30.7, 31.0, 31.0, 76.6, 76.9, 77.2,77.4, 121.9, 122.3, 122.4, 122.6, 125.0, 126.6, 127.3, 127.8, 127.9, 128.8, 128.9,131.6, 131.7, 132.3, 133.3, 133.6, 135.7, 136.9, 137.1, 139.7, 155.1, 155.2, 157.9, 162.3, 170.8$\delta$ ppm; MS (ESI+): *m/z* (%) = 715.3 (100) $[M+H]^+$ calcd for $C_{41}H_{46}O_0Br$: 713.2478; found: 713.2479

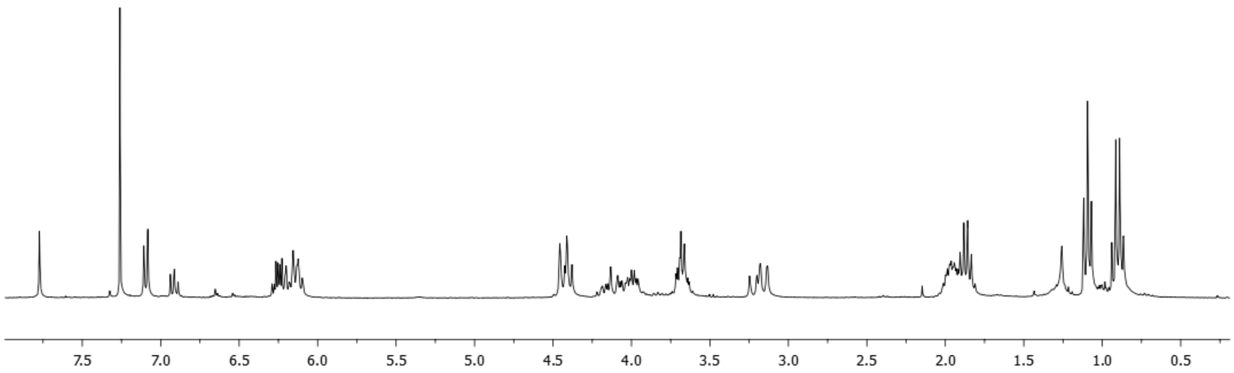


**Figure 25 – $^{1}$H NMR: (*cR*)-4-Bromo-carboxy-25,26,27,28-tetrapropoxycalix[4]arene**

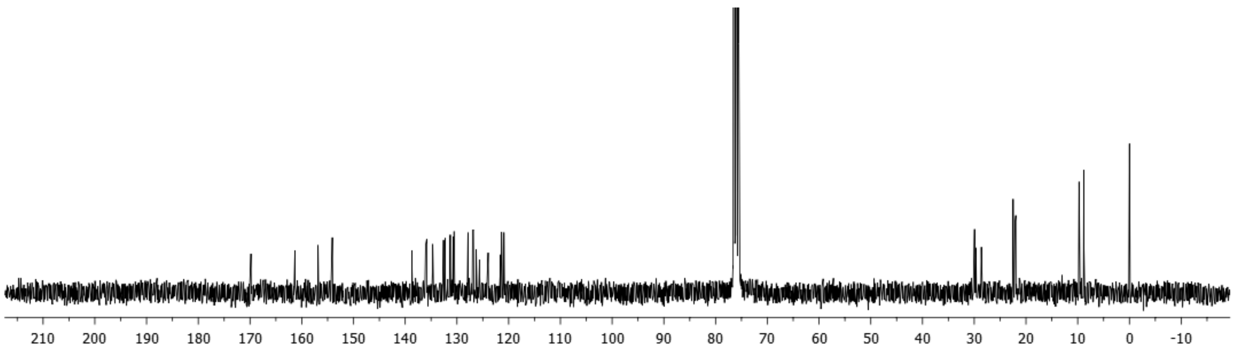


**Figure 26 - $^{13}$C NMR: (*cR*)-4-Bromo-carboxy-25,26,27,28-tetrapropoxycalix[4]arene**

# 6. References


(1) Herbert, S. A.; Arnott, G. E. *Org. Lett.* **2010**, *12*, 4600.

(2) Herbert, S. A.; Arnott, G. E. *Org. Lett.* **2009**, *11*, 4986.

(3) Rehländer, P. *Berichte der Dtsch. Chem. Gesellschaft* **1894**, *27*, 2154.

(4) George, B.; Papadopoulos, E. P. *J. Org. Chem.* **1977**, *42*, 441.

(5) Constantine, R. N.; Kim, N.; Bunt, R. C. *Org. Lett.* **2003**, *5*, 2279.

(6) Trost, B. M.; Murphy, D. J. *Organometallics* **1985**, *4*, 1143.

(7) Klebe, J. F.; Finkbeiner, H.; White, D. M. *J. Am. Chem. Soc.* **1966**, *88*, 3390.

(8) Auburn, P. R.; Mackenzie, P. B.; Bosnich, B. *J. Am. Chem. Soc.* **1985**, *107*, 2033.

(9) You, S.-L.; Hou, X.-L.; Dai, L.-X.; Yu, Y.-H.; Xia, W. *J. Org. Chem.* **2002**, *67*, 4684.
